# Gastroenteritis Therapies in Developed Countries: Systematic Review and Meta-Analysis

**DOI:** 10.1371/journal.pone.0128754

**Published:** 2015-06-15

**Authors:** Stephen B. Freedman, Dion Pasichnyk, Karen J. L. Black, Eleanor Fitzpatrick, Serge Gouin, Andrea Milne, Lisa Hartling, Pediatric Emergency Research Canada Gastroenteritis Study Group

**Affiliations:** 1 Sections of Pediatric Emergency Medicine and Gastroenterology, Alberta Children’s Hospital, Alberta Children’s Hospital Research Institute, Cumming School of Medicine, University of Calgary, Calgary, Alberta, Canada; 2 Alberta Research Centre for Health Evidence, Department of Pediatrics, University of Alberta, Edmonton, Alberta, Canada; 3 Division of Pediatric Emergency Medicine, BC Children’s Hospital, University of British Columbia, Vancouver, British Columbia, Canada; 4 IWK Health Centre, Emergency Department, Department of Emergency Medicine, Dalhousie University, Halifax, Nova Scotia, Canada; 5 Section of Pediatric Emergency Medicine, Centre Hospitalier Universitaire Ste-Justine, Université de Montréal, Montréal, Quebec, Canada; Nottingham University, UNITED KINGDOM

## Abstract

**Context:**

Gastroenteritis remains a leading cause of childhood morbidity.

**Objective:**

Because prior reviews have focused on isolated symptoms and studies conducted in developing countries, this study focused on interventions commonly considered for use in developed countries. Intervention specific, patient-centered outcomes were selected.

**Data Sources:**

MEDLINE, EMBASE, the Cochrane Database of Systematic Reviews, trial registries, grey literature, and scientific meetings.

**Study Selection:**

Randomized controlled trials, conducted in developed countries, of children aged <18 years, with gastroenteritis, performed in emergency department or outpatient settings which evaluated oral rehydration therapy (ORT), antiemetics, probiotics or intravenous fluid administration rate.

**Data Extraction:**

The study was conducted in accordance with the Cochrane Handbook for Systematic Reviews of Interventions and the PRISMA guidelines. Data were independently extracted by multiple investigators. Analyses employed random effects models.

**Results:**

31 trials (4,444 patients) were included. *ORT*: Compared with intravenous rehydration, hospitalization (RR 0.80, 95%CI 0.24, 2.71) and emergency department return visits (RR 0.86, 95%CI 0.39, 1.89) were similar. *Antiemetics*: Fewer children administered an antiemetic required intravenous rehydration (RR 0.40, 95%CI 0.26, 0.60) While the data could not be meta-analyzed, three studies reported that ondansetron administration does increase the frequency of diarrhea. *Probiotics*: No studies reported on the primary outcome, three studies evaluated hospitalization within 7 days (RR 0.87, 95%CI 0.25, 2.98). *Rehydration*: No difference in length of stay was identified for rapid vs. standard intravenous or nasogastric rehydration. A single study found that 5% dextrose in normal saline reduced hospitalizations compared with normal saline alone (RR 0.70, 95% CI 0.53, 0.92).

**Conclusions:**

There is a paucity of patient-centered outcome evidence to support many interventions. Since ORT is a low-cost, non-invasive intervention, it should continue to be used. Routine probiotic use cannot be endorsed at this time in outpatient children with gastroenteritis. Despite some evidence that ondansetron administration increases diarrhea frequency, emergency department use leads to reductions in intravenous rehydration and hospitalization. No benefits were associated with ondansetron use following emergency department discharge.

## Introduction

Gastroenteritis results in nearly 2 million pediatric emergency department (ED) visits in the United States annually.[1] Although rotavirus vaccination has altered the epidemiology of acute gastroenteritis (AGE),[2] emerging pathogens such as norovirus[3] continue to result in symptoms prompting medical evaluation.[4]

While systematic reviews (SR) have evaluated treatment options,[5–11] they are often inconclusive or discordant. Recent guidelines published by European Society for Pediatric Gastroenterology, Hepatology and Nutrition (EPGHAN) and the European Society of Pediatric Infectious Diseases (ESPID)[12] and those issued by the National Institute for Health and Clinical Excellence (NICE)[13] are vague regarding their recommendation regarding the use of antiemetics, a therapy endorsed by other position papers and meta-analyses.[10,11,14] The aforementioned guidelines also have differing recommendations regarding probiotics (Table 1).[15] The most recent guidelines endorsed by the American Academy of Pediatrics were published over a decade ago.[16]

**Table 1:** Summary of differing recommendations of prominent gastroenteritis guidelines.

	Antiemetics	Probiotics
Guarino A, Ashkenazi S, Gendrel D, Lo Vecchio A, Shamir R, Szajewska H European society for pediatric gastroenterology, hepatology, and nutrition/european society for pediatric infectious diseases evidence-based guidelines for the management of acute gastroenteritis in children in europe: update 2014. *J Pediatr Gastroenterol Nutr* 2014, 59:132–152.	Ondansetron, at the dosages used in the available studies and administered orally or intravenously, may be effective in young children with vomiting related to AGE. **Before a final recommendation is made, a clearance on safety in children is, however, needed** (II, B) (strong recommendation, low-quality evidence).	**Administration of effective probiotic strains reduce the duration of hospital stay and may be considered in children admitted with AGE** (II, B) (strong recommendation, low quality evidence). Active treatment with probiotics, in adjunct to ORS, is effective in reducing the duration and intensity of symptoms of gastroenteritis. Selected probiotics can be used in children with AGE (I, A) (strong recommendation, moderate-quality evidence). New evidence has confirmed that probiotics are effective in reducing the duration of symptoms in children with AGE (I, A) (strong recommendation, moderate-quality evidence). **The use of the following probiotics should be considered in the management of children with AGE** as an adjunct to rehydration therapy: L rhamnosus GG and S boulardii (I, A) (strong recommendation, low-quality evidence).
National Collaborating Centre for Women's and Children's Health Diarrhoea and vomiting caused by gastroenteritis: diagnosis, assessment and management in children younger than 5 years. Commissioned by the National Institute for Health and Clinical Excellence; Available at: http://www.nice.org.uk/guidance/cg84/resources/cg84-diarrhoea-and-vomiting-in-children-under-5-full-guideline2. Accessed October 15, 2014.	The guideline development group (GDG) considered that evidence from randomised controlled trials indicated that oral ondansetron could increase the success rate with oral rehydration therapy. The GDG was concerned that ondansetron might have adverse effects such as worsening diarrhoea. There was no evidence to support other agents, including metoclopramide and dexamethasone. **The GDG concluded that administration of anti-emetics could not currently be recommended.**	There was evidence from a high-quality systematic review suggesting that probiotic treatment had a beneficial effect–shortening the duration of diarrhoea and reducing the stool frequency. However, the available studies varied in quality, in the specific probiotics studied, in the treatment regimens used and in the outcomes examined. Therefore, despite some evidence of possible clinical benefit, **the GDG did not consider it appropriate to recommend the use of a probiotic at this time.**
Cheng A, Canadian Paediatric Society—Acute Care Committee Emergency department use of oral ondansetron for acute gastroenteritis-related vomtiing in infants and children. *Paediatr Child Health* 2011, 16:177–179.	**Oral ondansetron therapy, as a single dose, should be considered for infants and children six months to 12 years of age** who present to the ED with vomiting related to suspected acute gastroenteritis, and who have mild to moderate dehydration or who have failed oral rehydration therapy.	Not applicable.
Piescik-Lech M, Shamir R, Guarino A, Szajewska H Review article: the management of acute gastroenteritis in children. *Aliment Pharmacol Ther* 2013, 37:289–303.	New evidence indicates that **ondansetron, at the dosages used in the studies and administered orally or intravenously, may be considered for use in young children** with vomiting related to AGE. However, before a final recommendation is made, a clearance on safety in children is needed.	**New evidence has confirmed that the probiotics currently supported by ESPGHAN/ESPID–Lactobacillus GG and S. boulardii–are effective in reducing the duration of diarrhoea**. Current evidence clearly indicates that these are not the only effective probiotic microorganisms; however, these are the most studied. Probiotic effects are strain-specific, so the efficacy and safety of each should be established.
King CK, Glass R, Bresee JS, Duggan C Managing acute gastroenteritis among children: oral rehydration, maintenance, and nutritional therapy. *MMWR Recomm Rep* 2003, 52:1–16.	No clear recommendation in report.	Ondansetron, a serotonin antagonist, either by the oral or IV route, can be effective in decreasing vomiting and limiting hospital admission. However, reliance on pharmacologic agents shifts the therapeutic focus away from appropriate fluid, electrolyte, and nutritional therapy, can result in adverse events, and can add unnecessarily to the economic cost of illness. Because acute diarrhea is a common illness, **cost-effective analyses should be undertaken before routine pharmacologic therapy is recommended.**

Participant eligibility criteria in 'gastroenteritis' studies can vary significantly between studies. Since the diagnosis is made on clinical grounds, the precision is debatable. Most clinical trials employ clearly defined clinical features to determine eligibility. As these studies reflect pragmatic considerations and employ randomization, they retain internal validity. However, without careful consideration, their grouping together in SR and meta-analyses can be problematic. Consequently, significant variation has been documented in the management of AGE in developed countries at institutional,[17] national,[18] and international levels.[19]. This is in part explained by heterogeneity–population, setting, etiologic agents, and nutritional status. Studies from low and middle income countries include more severe cases, organisms rarely seen in developed nations, and malnourished children.[20,21] Outcome selection is increasingly a concern with most SRs focusing on diarrhea duration–a single symptom which in addition to being heterogeneous itself, also overlooks other key symptoms (e.g. vomiting). An analysis of 138 pediatric AGE randomized clinical trials (RCT) identified 64 unique definitions of diarrhea, and 69 of “resolution.”[22]

Driven by recent evidence and uncertainties in practice, the efficacy of oral rehydration therapy (ORT), antiemetics, probiotics and intravenous rehydration in developed countries was evaluated.

## Methods

A protocol (**S1 File**) was established a priori and followed standard SR procedures.[23] The planned approach involved initially conducting a comprehensive search to identify all relevant SRs performed to date to ensure that previously identified relevant studies were included in the review. This was followed by a thorough search for additional studies. Lastly, the evidence focusing on studies of outpatient children in developed countries was re-examined.

### Information Sources and Searches

A medical librarian (A.M.) developed the search strategy in collaboration with the research team to identify previous SRs of: (1) ORT; (2) antiemetics; (3) probiotics; and (4) intravenous fluid therapy (IVT). The following sources were searched: (a) MEDLINE (2000 to April 2012), EMBASE (2000 to April 2012), and the Cochrane Database of Systematic Reviews (2005 to April 2012) via the OvidSP platform; (b) appropriate journals and major, relevant scientific meetings; (c) reference lists of relevant reviews; and (d) primary authors were contacted. The search was not restricted by language or publication status. All studies contained in previous relevant SRs were screened for inclusion. This approach is an economically efficient method of identifying all prior relevant studies dating back to the origins of the search engines.

The librarian then searched the literature to identify trials published since the dates included in the earliest SR identified. The search included electronic databases (i.e. MEDLINE, EMBASE, Cochrane Central Register of Controlled Trials; **S2 File**) and the grey literature. The latter search included clinical trials registries (clinicaltrials.gov, the World Health Organization trials registry, and the Current Controlled Trials registry) and conference proceedings (Society for Pediatric Research, American Academy of Pediatrics, Canadian Pediatric Society, International Conference on Emergency Medicine; 2010–2012). Reference lists were screened and experts contacted. No language restrictions were employed. The search was re-run in September 2014 to identify any recently published studies.

### Inclusion Criteria

Search results were screened independently by two reviewers to identify potentially relevant citations. The full text of potentially relevant citations was assessed for inclusion by two independent reviewers using predefined criteria. Disagreements were resolved by consensus. Eligible RCTs involved children <18 years of age with AGE and evaluated: (1) any ORT regimen vs. intravenous or nasogastric rehydration; (2) any antiemetic medication vs. placebo or alternative; (3) any probiotic agent vs. placebo or alternative; and (4) different rates and compositions of intravenous fluid rehydration protocols. Studies were included if the condition evaluated was consistent with AGE and the location was an ED or similar outpatient setting in a developed nation as defined by the United Nations (i.e. Australia, Canada, European countries, Japan, New Zealand, and the United States).[24]

### Outcomes

The interventions and outcome measures were identified by clinician authors (SF, KB, EF, SG) and knowledge users (DJ, FB, BH, MJ, TK) a priori based on clinical relevance incorporating recommendations to employ outcomes of interest to parents, clinicians, and health systems (Table 2).[25]

**Table 2:** Interventions and their specific outcomes evaluated.

Primary Outcome	Secondary Outcomes
**Oral Rehydration Therapy**	
Hospitalization	Length of Stay
	Return Visits
	Adverse Effects
**Antiemetic Agents**	
Administration of Intravenous Rehydration	Hospitalization
	Length of Stay
	Return Visits
	Adverse Effects
**Probiotic Agents**	
Any Subsequent Healthcare Visit (7 days)	Administration of Intravenous Rehydration
	Hospitalization
	Adverse Effects
**Intravenous Fluid Therapy**	
Length of Stay	Hospitalization
	Return Visits
	Dysnatremia*

*The term dysnatremia refers to the presence of a serum sodium value outside of the age accepted range of normal values.

### Data Extraction

As is commonly performed, one reviewer extracted data using a structured form with verification performed by a second reviewer.[26–29] Items extracted were: study characteristics, participants, interventions and comparisons, outcomes, funding source, and results. Data were entered into Microsoft Excel (Microsoft, Redmond, WA). Disagreements were resolved by consensus, or by a third reviewer.

### Risk of Bias Assessment

The Cochrane Risk of Bias (RoB) tool[23] was applied, independently by two reviewers to assess internal validity (**S1 Table**). Discrepancies were resolved by consensus or by involving a third reviewer.

### Grading the Body of Evidence

The quality of evidence was assessed using methods developed by the GRADE Working Group.[30] For each comparison and outcome, the following were assessed: risk of bias, consistency, directness, and precision. Overall quality was graded as high, moderate, low, or very low by two reviewers with discrepancies resolved through discussion.

### Statistical Analysis

Evidence tables to describe the studies were developed. A quantitative analysis synthesized numerically the effectiveness of each intervention and investigated heterogeneity. Data was reported for continuous outcomes as mean differences which were combined, where appropriate, using a weighted mean difference and inverse-variance methods.[31] Data for dichotomous outcomes are reported employing risk ratios (RR) or risk differences. The latter was employed when there were zero events in one of the treatment arms of an individual study which contributed data to the meta-analysis (e.g. adverse events). Results are reported with 95% confidence intervals. The primary analysis was based on a random effects model due to anticipated clinical variability between studies.[32] Sensitivity analyses were conducted using a fixed effects model and no differences were identified in the results. Heterogeneity was quantified using the I-squared statistic.[33,34] When heterogeneity was substantial (I^2^ ≥75%), pooling of studies was not performed. Due to insufficient numbers pre-planned sensitivity analyses based on risk of bias, intention-to-treat analysis, and funding source could not be conducted. Testing for publication bias was not performed due to insufficient numbers. Analyses were conducted using Review Manager 5.0 (The Cochrane Collaboration, Copenhagen, Denmark).

## Results

**Fig 1:**
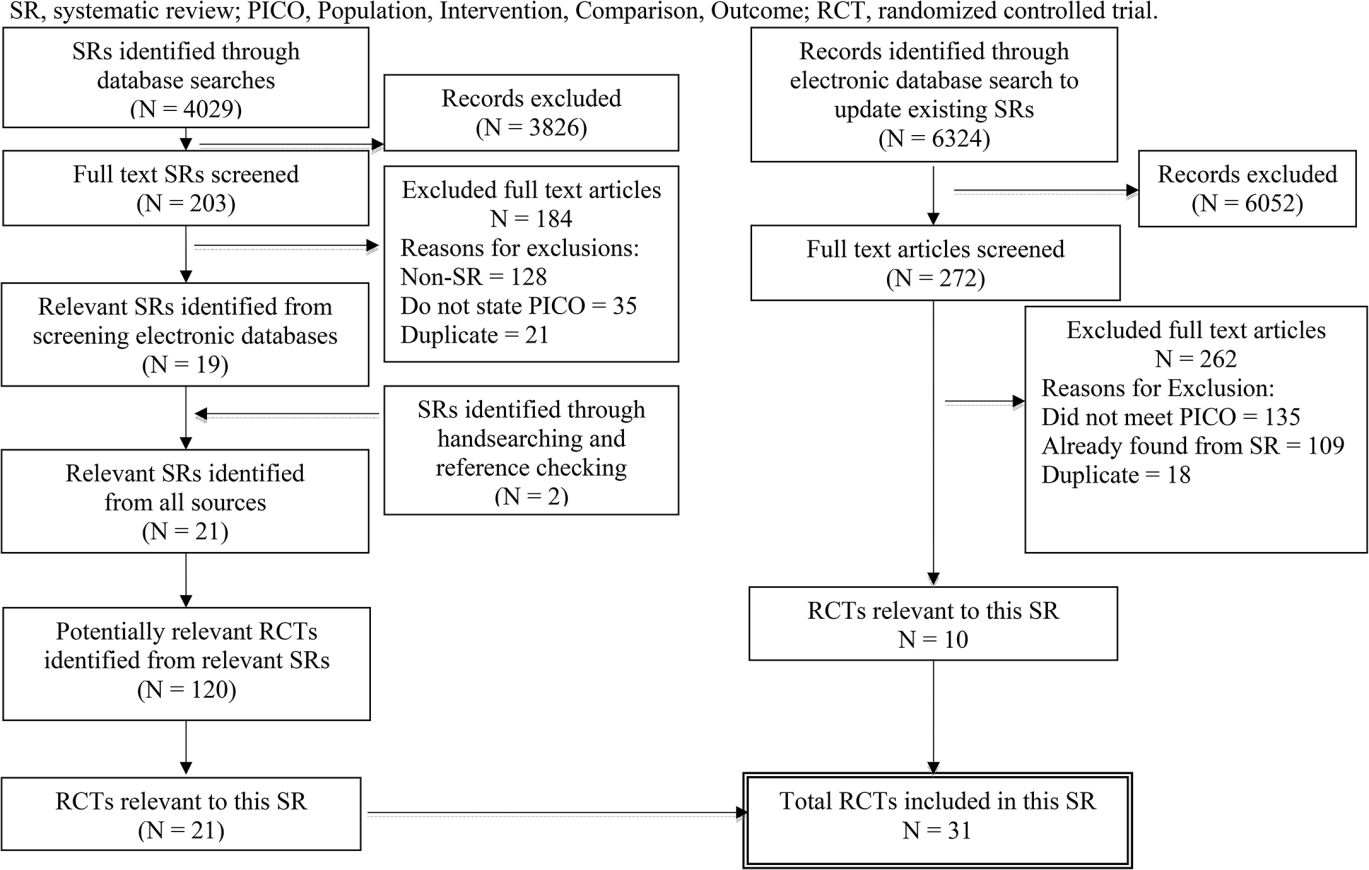
Flow diagram of study selection. SR, systematic review; PICO, Population, Intervention, Comparison, Outcome; RCT, randomized controlled trial.

Sixty-six RCTS were relevant; 35 did not report any of the a priori identified outcomes of interest, therefore, 31 RCTs involving 4,444 patients were included (Fig 1; Table 3). Four antiemetic agents were studied along with 11 different probiotic strains. Overall risk of bias was low for 23% of trials (7/31), unclear for 74% (23/31), and high for 3% (1/31); **S1 Table**. Industry funding was identified in the following studies: ORT – 5 (50%); antiemetics – 3 (38%); probiotics – 2 (33%); IVT – 2 (33%).

**Table 3:** Overview of studies included in systematic review.

Comparison	Number of studies (Number of patients)	Number of studies providing data for primary outcome (Number of patients)	Number of studies providing data for secondary outcomes (Number of patients)	Years of publication, median (range)	Countries of study (Numer of studies)	Risk of bias
Intravenous Therapy vs. Oral Rehydration Therapy	10 (599)	3 (136)	10 (599)	1992 (1985–2005)	Australia (1), Canada (1), Finland (1), USA (7)	9 unclear, 1 low
Any Antiemetic vs. Placebo	9 (1691)	7 (1043)	9 (1691)	2008 (2002–2014)	Australia (1), Canada (1), Germany (1), Saudi Arabia (1), USA (5)	7 unclear, 2 low
Any Probiotic vs. Placebo	6 (1170)	1 (155)	6 (1170)	2009 (2007–2012)	Australia (1), Italy (2),Ukraine (2), USA (1)	4 unclear, 2 low
Intravenous Fluid Rates & Compositions	6 (984)	2 (305)	4(644)	2011 (2006–2014)	Australia (2), Canada (1), USA (1)	3 unclear, 2 low, 1 high

### Oral Rehydration Therapy (ORT)

Ten studies involving 599 patients compared ORT with IVT (**S2 Table**). Sample sizes ranged from 24 to 111 (median 47, inter-quartile range 35 to 91). Five studies did not report dehydration severity; the remainder, with one exception,[35] included primarily children with mild dehydration.

**Fig 2:**
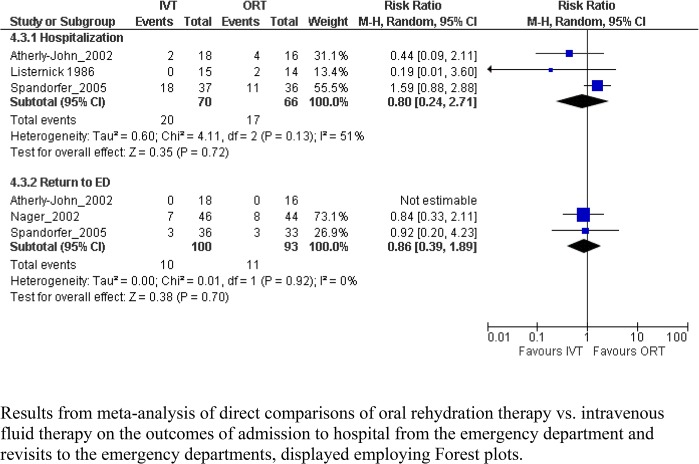
Meta-graph comparing oral rehydration therapy vs. intravenous fluid therapy. Results from meta-analysis of direct comparisons of oral rehydration therapy vs. intravenous fluid therapy on the outcomes of admission to hospital from the emergency department and revisits to the emergency departments, displayed employing Forest plots.

Three studies provided data on the effect for the primary outcome of hospitalization, which in meta-analysis, showed no significant difference between groups (RR 0.80, 95% CI 0.24, 2.71, I^2^ = 51%; Table 4; Fig 2). The quality of evidence was low owing to inconsistencies in effect estimates across studies and imprecision in the pooled result. No difference was observed in the secondary outcome of return to the ED. Six studies provided data on ED length of stay. However, there was substantial heterogeneity across studies and results could not be pooled (I^2^ = 91%). Quality of the evidence was very low. The mean difference in length of stay reported by individual studies, including time spent in hospital, ranged from 1.20 days less for ORT (95% CI -2.16, -0.24) to 0.92 days longer (95% CI 0.31, 1.53). Five studies reported on adverse effects (Table 5); no significant differences were identified.

**Table 4:** Results for Primary and Secondary Outcomes.

Comparison	Outcome	Number of studies (Number of patients)	Risk Ratio (95% CI)	I^2^ (%)	Quality of evidence based on GRADE
**Oral Rehydration Therapy**					
**IVT vs. ORT**					
**Primary**	Hospitalization	3 (136)	0.80 (0.24, 2.71)	51	Low
**Secondary**	Length of ED Stay	6 (308)	Not pooled due to substantial heterogeneity	91	very low
	Return to ED	3 (193)	0.86 (0.39, 1.89)	0	moderate
**Antiemetics**					
**Any antiemetic vs. placebo**					
**Primary**	IV Fluid Administration	5 (733)	0.40 (0.26, 0.60)†	30	High
**Secondary**	Hospitalization	7 (1043)	0.44 (0.23, 0.82)†	27	High
	Return to ED	8 (1074)	1.31 (0.73, 2.35)	52	moderate
	Length of ED Stay	5 (991)	Not pooled due to substantial heterogeneity	75	moderate
**Dimenhydrinate vs. placebo**					
**Primary**	IV Fluid Administration	1 (144)	0.74 (0.29, 1.87)	NA	low
**Secondary**	Hospitalization	2 (368)	0.72 (0.34, 1.53)	0	moderate
	Return to ED	2 (343)	0.61 (0.34, 1.12)	0	moderate
**Ondansetron vs. dexamethasone**					
**Primary**	Hospitalization	1 (93)	0.29 (0.06, 1.33)	NA	low
**Secondary**	Return to ED	1 (56)	4.30 (1.00, 18.47)†	NA	low
**Ondansetron vs. placebo**					
**Primary**	IV Fluid Administration	3 (433)	0.38 (0.27, 0.54)†	0	High
**Secondary**	Hospitalization	5 (613)	0.32 (0.18, 0.57)†	2	moderate
	Return to ED	5 (609)	1.57 (0.70, 3.52)	36	Low
	Length of ED Stay	4 (826)	Not pooled due to substantial heterogeneity	NA	moderate
**Granisetron vs. placebo**					
**Primary**	IV Fluid Administration	1 (156)	0.05 (0.00, 0.78)	NA	Very low
**Secondary**	Hospitalization	1 (165)	3.04 (0.13, 73.46)	NA	Very low
	Return to ED	1 (122)	3.09 (1.19, 8.05)	NA	Very low
	Length of ED Stay	1 (165)	-0.65 (-1.29, -0.01)	NA	Very low
**Probiotics**					
**Probiotic (Any) vs. Placebo**					
**Primary**	Return to ED	1 (155)	0.78 (0.36, 1.67)	NA	Low
**Secondary**	Hospitalization	3 (833)	0.53 (0.26, 1.07)	20	Low
	IV Fluid Administration	1 (64)	1.13 (0.81, 1.57)	NA	Low
**Probiotic (Combo) vs. Placebo**					
**Secondary**	Hospitalization	1 (189)	0.47 (0.09, 2.53)	NA	very low
***B*. *clausii* vs. Placebo**					
**Secondary**	Hospitalization	1 (192)	0.92 (0.24, 3.57)	NA	very low
***E*. *faecium* vs. Placebo**					
**Secondary**	Hospitalization	1 (183)	1.01 (0.26, 3.92)	NA	very low
***L*. *casei* vs. Placebo**					
**Secondary**	IV Fluid Administration	1 (64)	1.13 (0.81, 1.57)	NA	Low
***L*. *paracasei* vs. Placebo**					
**Secondary**	Hospitalization	1 (107)	0.37 (0.18, 0.75)†	NA	Low
***L*. *rhamnosus* vs. Placebo**					
**Primary**	Return to ED	1 (155)	0.78 (0.36, 1.67)	NA	very low
**Secondary**	Hospitalization	2 (347)	0.65 (0.08, 5.45)	43	very low
***S*. *boulardii* vs. Placebo**					
**Secondary**	Hospitalization	1 (183)	1.01 (0.26, 3.92)	NA	very low
**IV Fluids**					
**Rapid IV vs. Standard IV**					
**Primary**	Length of ED Stay >6 hours	1 (226)	1.06 (0.74, 1.53)	NA	low
**Secondary**	Hospitalization	2 (318)	1.03 (0.44, 2.39)	23	low
	Return to ED	2 (311)	0.88 (0.52, 1.48)	0	low
**Rapid NG vs. Standard NG**					
**Primary**	Length of ED Stay*	1 (228)	-1.90 (-9.11, 5.31)	NA	very low
**Secondary**	Hospitalization	1 (228)	0.69 (0.49, 0.97)†	NA	low
**Isotonic IV Fluid vs. Hypotonic IV Fluid**					
**Secondary**	Dysnatremia	1 (44)	-0.23 (-0.41, -0.04)†	NA	very low
**5% Dextrose in Normal Saline vs. Normal Saline**					
**Primary**	Length of ED Stay	1 (188)	0.13 (-0.27, 0.53)	NA	Very low
**Secondary**	Hospitalization	1 (114)	0.70 (0.53, 0.92)	NA	Very low
	Return visits	1 (80)	0.64 (0.30, 1.36)	NA	Very low
**Plasma-Lyte A vs. 0.9% Sodium Chloride**					
**Secondary**	Hospitalization	1 (54)	1.09 (0.59, 2.03)	NA	Very low

IVT, Intravenous Therapy; ORT, Oral Rehydration Therapy; ED, Emergency Department; GRADE, Grading of Recommendations Assessment, Development and Evaluation; IV, intravenous; NA, Not Applicable; NG, nasogastric; vs, versus.

† Statistically significant effect.

*Length of stay, as a continuous variable, is reported as mean difference (95% CI).

**Table 5:** Adverse Events.

Adverse Event	Total number of patients	Number of events/total (%)	Risk Difference (95% CI)	Risk Ratio* (95% CI)	I^2^ (%)‡
**IVT vs ORT**					
Periorbital Edema	219	IVT 6/99 (6); ORT 8/120 (7)	0.03 (-0.07, 0.13)	1.30 (0.29, 5.87)	54
Hyponatremia	104	IVT 3/52 (6); ORT 4/52 (8)	0.02 (-0.08,0.12)	1.33 (0.31, 5.67)	NA
Seizure	152	IVT 1/67 (1); ORT 1/85 (1)	0.00 (-0.07, 0.08)	0.70 (0.04, 11.94)	31
Phlebitis	52	IVT 0/17 (0); ORT 0/35 (0)	0.00 (-0.08, 0.08)	-	NA
**Antiemetics**					
Headache	137	Dimenhydrinate 3/69 (4); Placebo 1/68 (2)	0.03 (-0.03, 0.08)	2.96 (0.32, 27.72)	NA
Rash	137	Dimenhydrinate 4/69 (6); Placebo 0/68 (0)	0.06 (0.00, 0.12)	-	NA
Hyperactivity	137	Dimenhydrinate 4/69 (6); Placebo 0/68 (0)	0.06 (0.00, 0.12)	-	NA
GI Upset	137	Dimenhydrinate 3/69 (4); Placebo 3/68 (3)	0.00 (-0.07, 0.07)	0.99 (0.21, 4.71)	NA
Sedation	208	Dimenhydrinate 22/106 (22); Placebo 18/102 (19)	0.03 (-0.08, 0.14)	1.18 (0.67, 2.06)	NA
Exanthem	208	Dimenhydrinate 1/106 (1); Placebo 1/102 (1)	0.00 (-0.03, 0.03)	0.96 (0.06, 15.18)	NA
Drowsiness	137	Dimenhydrinate 29/69 (46); Placebo 25/68 (37)	0.01 (-0.02, 0.22)	1.14 (0.75, 1.73)	0
Urticaria	214	Ondansetron 0/107 (0); Placebo 1/107 (1)	-0.01 (-0.03, 0.02)	-	NA
**Probiotics**					
Rhinitis	113	Escherichia coli Nissle 1/55 (2); Placebo 0/58 (0)	0.02 (-0.03, 0.07)	-	NA
Otitis Media	113	Escherichia coli Nissle 1/55 (2); Placebo 0/58 (0)	0.02 (-0.03, 0.07)	-	NA
Abdominal Pain	261	Escherichia coli Nissle 2/130 (1); Placebo 4/131 (3)	0.01 (-0.07, 0.05)	0.67 (0.06, 7.97)	60
Hypersensitivity	151	Escherichia coli Nissle 1/75 (1); Placebo 0/76 (0)	0.01 (-0.02, 0.05)	-	NA
**Isotonic IV Fluids vs Hypotonic IV Fluids**					
Dysnatremia	44	Isotonic solution 0/20 (0); Hypotonic solution 5/22 (23)	-0.23 (-0.41, -0.04)†	-	NA
**Plasma-Lyte A vs. 0.9% Sodium Chloride**					
Dysnatremia	75	Plasma-Lyte A 1/38 (3)0.9% Sodium Chloride 1/37 (3)	0.00 (-0.07, 0.07)	0.97 (0.06, 15.00)	NA

IVT, Intravenous Therapy; ORT, Oral Rehydration Therapy; GI, Gastrointestinal; IV, Intravenous; NA, Not Applicable.

* Risk ratio calculated where there was at least one incidence in each group

† Statistically significant difference between groups

‡ Based on risk difference

### Antiemetics

**Fig 3:**
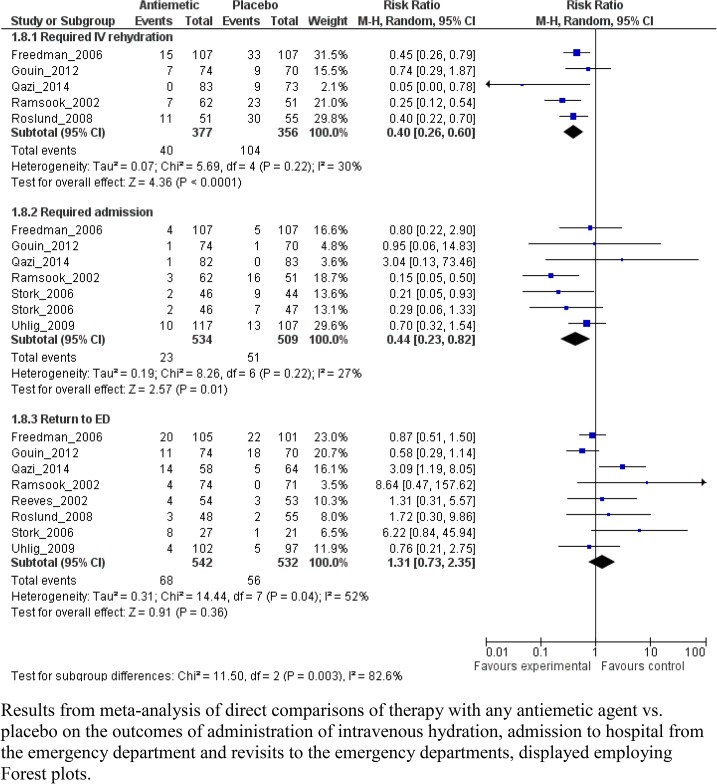
Meta-graph comparing any antiemetic therapy vs. placebo. Results from meta-analysis of direct comparisons of therapy with any antiemetic agent vs. placebo on the outcomes of administration of intravenous hydration, admission to hospital from the emergency department and revisits to the emergency departments, displayed employing Forest plots.

Nine studies involving 1,691 patients evaluated three antiemetic agents: ondansetron (N = 6), dimenhydrinate (N = 2), and granisetron (N = 1). Eight RCTs compared the antiemetic agent with placebo; one RCT compared ondansetron with dexamethasone (Table 3; Fig 3; **S3 Table**).

All five studies demonstrated a reduction in the primary outcome of intravenous rehydration usage amongst children administered an antiemetic agent (Fig 3; RR 0.40, 95% CI 0.26, 0.60, I^2^ = 30%; N = 733). Quality of evidence for this outcome was high. Pooled results demonstrated that patients receiving an antiemetic agent were hospitalized less often (RR 0.44, 95% CI 0.23, 0.82, I^2^ = 27%; N = 1043). There was substantial heterogeneity for length of stay results across studies (I^2^ = 75%); therefore, data were not pooled. Mean length of stay reported in individual studies ranged from 0.23 hours (95% CI -0.49, 0.03) to 1.0 hours (95% CI -1.34, -0.66) less for antiemetics. There was no difference between groups in the proportions of children experiencing ED revisits.

Findings were similar when only those studies involving ondansetron were analyzed. No significant differences were identified when studies involving dimenhydrinate were evaluated. Three studies evaluating dimenhydrinate reported specific adverse events—drowsiness, headache, rash, hyperactivity, gastrointestinal upset, and sedation; no differences were noted compared with placebo (Table 5).

Diarrhea frequency was evaluated in several studies but due to the varying methods of reporting the findings, the results could not be combined. Following a single dose of ondansetron or placebo, during ED ORT one study (N = 215) reported that ondansetron administration resulted in a statistically significant increase in diarrhea frequency (1.4 vs. 0.5 stools; group (P < 0.001).[36] Another study, which similarly evaluated single oral dose ondansetron vs. placebo, collected post-discharge diarrhea frequency information. In their cohort (N = 106), the median number of episodes of diarrhea post-discharge was 0 in both groups; 93% of children administered placebo and 80% of those administered ondansetron had < 3 episodes of diarrhea after discharge and the mean was 1.8 vs. 0.5 episodes of diarrhea respectively (no test of significance provided).[37] A multi-dose ondansetron vs. placebo study (N = 145) reported no difference in stool frequency while in the ED (mean 0.70 vs. 0.61 episodes respectively; P = 0.62); however, following discharge there was a significant increase in stool frequency amongst those administered ondansetron at both 24 (4.7 vs. 1.4; P = 0.002) and 48-hour (3.0 vs. 1.0; P = 0.02) outcome time points.[38] A study evaluating single dose, intravenous ondansetron (N = 107), reported that compared with placebo, there were no significant differences in the proportion (41%—ondansetron; 40%—placebo; P = 0.93), frequency (median of 5 in both groups; P = 0.87) or duration (60 hours in the ondansetron vs. 49 hours in placebo groups; P = 0.72) of diarrheal episodes following the intervention.[39] Lastly, a multi-dose study of granisetron (N = 165) reported a similar odds (OR 1.29, 95% CI 0.64, 2.60) and frequency of diarrhea following medication administration (6.5 ± 6.1 vs. 5.8 ± 7.6; P = 0.51).[40]

### Probiotics

Six studies, involving 1170 patients, examined different probiotics (Table 4; **S4 Table**); five compared individual probiotic agents with placebo, one compared multiple and combination products with placebo.

**Fig 4:**
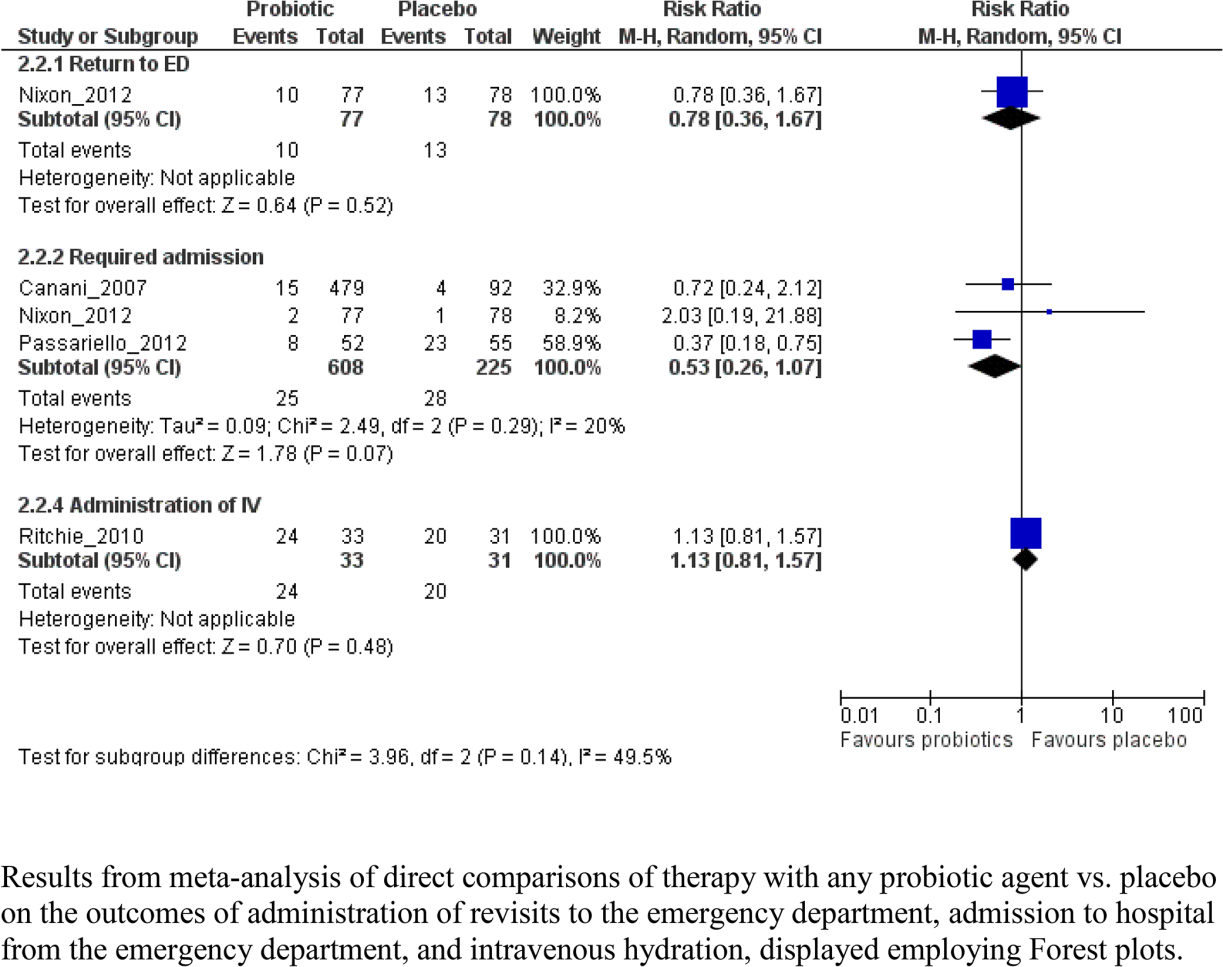
Meta-graph comparing any probiotic therapy vs. placebo. Results from meta-analysis of direct comparisons of therapy with any probiotic agent vs. placebo on the outcomes of administration of revisits to the emergency department, admission to hospital from the emergency department, and intravenous hydration, displayed employing Forest plots.

No studies reported findings related to any subsequent healthcare provider visits. One study reported no difference between groups in terms of return for additional ED care (Fig 4 and Table 4). Pooled results from 3 studies showed no difference between groups for hospitalization within 7 days (RR 0.53, 95% CI 0.26, 1.07, I^2^ = 20%; N = 571). Based on one study, no difference was observed between probiotic and placebo groups in the need to administer intravenous rehydration within 7 days.

When analyzed by individual probiotic product, most comparisons included a single RCT and reported no significant differences between groups. The quality of evidence for all comparisons was low or very low. Specific adverse events were reported in only one study; no differences were found between groups (Table 5).

### Intravenous Fluid Therapy (IVT)

Six studies involving 984 patients compared different rates or compositions of intravenous fluids (**S5 Table**). Two studies (N = 318) compared rapid (60 ml/kg and 50 ml/kg over 1 hour) vs standard rehydration rates (20 ml/kg over 1 hour and 50 ml/kg over 3 hours, respectively); one study compared rapid polyelectrolyte with rapid nasogastric (NG) rehydration (N = 254); one study compared isotonic with hypotonic intravenous solutions (N = 124); one study compared 5% dextrose in normal saline solution with normal saline solution alone (N = 188); and, one study compared Plasma-Lyte A with 0.9% sodium chloride (N = 100).

No difference in length of stay was identified for rapid vs. standard IVT or rapid vs. standard NG rehydration (Table 4). Two studies comparing rapid vs. standard IVT also identified no difference between groups in admissions or ED revisits. Significantly fewer admissions occurred with rapid compared with standard NG rehydration. The study comparing isotonic vs. hypotonic IVT did not report on any of the outcomes of interest except for dysnatremia; significantly fewer cases occurred with isotonic intravenous hydration. A reduction in hospitalizations (RR 0.70, 95% CI 0.53, 0.92) was identified when 5% dextrose in normal saline was compared with normal saline; however, no differences were found for length of ED stay or return visits. Similarly, no differences were found for time to rehydration, hospitalization, or incidence of dysnatremia in the study comparing Plasma-Lyte A with 0.9% sodium chloride. Quality of evidence for all outcomes and comparisons was low or very low.

## Discussion

Key treatment decisions were examined—route of rehydration, use of antiemetics and probiotics, and methods of intravenous and nasogastric rehydration—in a single SR, with a unique focus on children in developed countries in order to provide information needed by clinician and knowledge users. Although, the use of antiemetics confers short term benefits in outpatients by reducing intravenous rehydration administration and hospitalization, no difference was identified in terms of ED revisits. Aside from individual studies which documented positive results, no other interventions evaluated were found to result in improved outcomes. This must be interpreted with caution because most often there were insufficient numbers of eligible studies evaluating the outcomes of interest.

The treatment of AGE was approached from a perspective which yielded a paucity of studies from developed countries that reported on the a priori identified outcome measures. Consequently the findings contradict those of prior reviews which endorse the use of probiotics based on their ability to reduce the mean duration of diarrhea (by 25 hours), the likelihood of diarrhea lasting ≥4 days (risk ratio 0.41; 0.32 to 0.53), and stool frequency on day #2 (mean difference 0.80 stools).[8] However, prior reviews included heterogeneous groups of children and the importance of the outcomes evaluated has been questioned.[22] Because of the methodological limitations of many of the trials included in prior reviews of probiotics, it is suggested that the evidence be viewed with caution.[41]

To minimize heterogeneity and maximize relevance the study focused on a well-defined population, key interventions, and clinically important outcomes. This differs from other reviews that “summarise the more recent data” and search only MEDLINE and The Cochrane Database of Systematic Reviews.[15] Although a recent overview of reviews reported similar findings, the methodologies, which differed significantly, resulted in the inclusion of different studies.[42] Other prior meta-analyses have not restricted their populations to developed regions and have struggled with the inclusion of studies with varying outcome measure definitions (e.g. duration of diarrhea).[22] These two characteristics differentiate this review and underlie the differences in the studies included compared with others.

### Oral Rehydration Therapy

Although ORT is the most fundamental and accepted treatment,[16] studies comparing ORT to IVT generally provided inadequate descriptions of the severity of dehydration, which drives treatment decisions.[16] The trials identified were small, often single-centre, and rarely reported sample size calculations.[43] Children with mild dehydration were the typical target population, reflecting the overuse of IVT in North America.[44] Although no difference in the primary outcome of hospitalization was identified, in the context of the comparison evaluated (ORT vs. IVT), this supports the use of ORT.

Although oral rehydration solution (ORS) use per se (i.e. we did not focus on the solution used) was not evaluated, the conceptual approach of ORT as opposed to IVT was evaluated. Since ORS remains the cornerstone of AGE management and is considered to be the one of the top medical discoveries of the 20^th^ century,[45] its effectiveness in children with moderate dehydration was not questioned. Given the paucity of studies identified in this review, to further reduce IVT use in children with moderate dehydration in developed countries, quality improvement studies documenting the keys to successful knowledge translation in the target environments, are needed.[46] Such studies have been called for to enhance the ability to translate research findings into clinical practice to maximize the use of evidence-based therapies.[47]

### Antiemetics

Despite limited endorsement in most practice guidelines,[16] this intervention included the largest number of children of the interventions included in this study. While the results favoured the use of antiemetics as it relates to short-term outcomes, no difference was identified in ED revisits. This finding is in keeping with the expectations of single dose use of a medication with relatively short half-life. Although prior reviews have been hesitant to recommend ondansetron use in light of concerns related to its arrhythmogenic potential,[15] recent evidence has reduced concerns related to single oral dose use in otherwise healthy children.[48] Although we could not meta-analyze the available data on the impact of antiemetics on diarrhea frequency, the data we report does seem to indicate that ondansetron administration does increase the frequency of diarrhea. The clinical relevance of this increase (range: 0.1–0.9 stools while undergoing ORT) in the ED is minimal as reflected by the clinically relevant outcomes of intravenous rehydration and admission which are both reduced amongst children administered ondansetron relative to placebo. Following discharge, there similarly appears to be an increase in the number of diarrheal episodes and this appears to be most pronounced with multi-dose therapy. Given the lack of benefit seen with multi-dose therapy and the increased risk of diarrhea, such regimens are not recommended,[49]

### Probiotics

European guidelines state that probiotics “should be considered in the management of children with AGE as an adjunct to rehydration therapy.”[12] Use in North America remains limited, and has been reported to be as low as 1% amongst inpatients in large U.S. academic pediatric centres.[50] Although over 60 studies have been conducted,[8] the current analysis raises concerns as it relates to outcomes evaluated. No studies evaluated the primary outcome identified as most important by our knowledge users—subsequent healthcare provider visits. This outcome was deemed to reflect a clinically significant benefit to the child and family and extends beyond simply measuring the absolute number of stools or time to last stool. Furthermore, the quality of the evidence included was 'very low' or 'low' and a disproportionate number of studies (4 out of 6) emerged from Italy and Ukraine.

This review grouped all probiotic products into a single intervention for analytical purposes. While not ideal, as not all probiotic preparations are equally effective,[51] it was necessary given the paucity of studies performed with each individual strain. Additionally, a prebiotic (nondigestible food that beneficially affects the host by selectively stimulating the growth/activity of colonic bacteria in the colon) plus probiotic (xilooligosaccharides plus arabinogalactan and Lactobacillus paracasei B21060)[52] study was included in the analyses. Sensitivity analyses, which were conducted when a minimum of two studies employing the same probiotic were identified, did not produce any changes in the conclusions.

A trend towards reductions in future hospitalizations was detected; although this did not achieve significance, one could interpret this as evidence of a possible clinical benefit associated with probiotic use. However, it is challenging to generalize findings from probiotic clinical trials with the most frequently studied strain (Lactobacillus GG) having its benefits confined to studies conducted outside North America.[41] The only North American outpatient study employing Lactobacillus GG found no difference in the time to normal stool or the number of diarrheal stools.[53]

### Intravenous Fluid Therapy

Few studies evaluating IVT were identified, and limited evidence supporting the use of rapid rehydration therapy was found. The limited evidence of benefit may relate to the inaccuracy of dehydration assessment[54] or a delay in the timing between intravascular rehydration and clinical improvement. As it relates to choice of maintenance IV hydration solution, a single, low quality study, reported that dysnatremias are more frequent amongst children administered hypotonic fluids.

### Overall

Investigators should conduct more sophisticated studies that answer clinically relevant questions employing outcomes of importance to end-users. Pragmatic, comparative effectiveness trials using factorial or non-inferiority designs and valid outcome measures[55] answering key questions such as the success of ORT in children with moderate dehydration would significantly enhance the uptake of ORT. Such work is needed to confirm and convince knowledge-users (if positive) of the utility of interventions such as probiotics. Although multiple meta-analyses have identified some benefits to be associated with the latter,[8,41] their uptake has been limited. Antiemetics have been investigated employing clinically important outcome measures and consequently uptake has occurred rapidly.[56] The use of patient-centered outcomes and well defined patient populations to minimize heterogeneity and maximize clinical applicability resulted in the exclusion of many reports which highlights the need for further investigations employing outcomes established as important to parents and children.[22] While diarrhea remains a concern with ondansetron administration the clinical impact appears to be negligible with single dose regimens, however when multiple doses are administered this becomes more of a concern.

This review has adhered to the latest methodological standards. Relevant evidence was searched for extensively and this review included all studies regardless of language of publication. Although not all possible interventions were considered, this review focused on common clinical intervention options and represents a comprehensive synthesis incorporating two key perspectives: 1) patient-centered outcomes, and 2) developed countries.

This review has several limitations. It is limited by the challenges of synthesizing unrelated outcomes which resulted in a limited body of evidence. Since this review focused on outpatient studies, the results cannot be applied to the care of children managed at home by caregivers for whom very limited data exists, or to the care of hospitalized children. The latter group, which represents a fraction of children with AGE, has been the focus of most clinical trials. While the ED setting may differ significantly from other outpatient settings and this might be a source of heterogeneity, within each therapy the location was very consistent (e.g. ondansetron studied in ED; probiotics studied in primary care). Since participant ages varied across the studies, planned sub-analyses (i.e. < 5 years vs. ≥ 5 years) could not be conducted. Additionally, the only interventions evaluated are those currently being considered for routine use in developed countries; interventions that are primarily considered for use only in developing nations (e.g. antibiotics, zinc) were not evaluated. Although "exp Diarrhea/” was included in the original search strategy (**S2 File**) data related to the duration and frequency of diarrhea was not abstracted originally as these were not included in the *a priori* defined outcome measures. However, in the context of antiemetic evaluation, they were deemed to be important and thus were included in the current version of the SR. Lastly, despite attempts to minimize heterogeneity amongst different studies, it could not be completely eliminated as studies included almost certainly varied in terms of infectious etiology, seasonality, and local factors influencing clinical decision making. Nonetheless, the knowledge gaps identified can serve to guide future research efforts.

## Conclusions

Although some clinical practice guidelines endorse probiotic use, there is a paucity of supporting evidence for their use in developed countries. Routine probiotic use appears unjustified at present and future studies employing patient-centered outcomes are needed. While further evidence supporting ORT is needed to expand its use, such studies may be challenging to justify as expert opinion overwhelmingly supports its use as first-line therapy in children with AGE. Ondansetron has a strong evidence base supporting use and the key will be ensuring that administration is directed at the populations included in the RCTs. It should be noted that ondansetron use is not associated with reduction in ED revisits and it has the potential to increase diarrheal episodes. Moving forward, studies focusing on important outcomes and patient populations are needed to build a stronger evidence base to guide therapy for this extremely common condition.

## Supporting Information

S1 File Protocol. (PDF)

S2 File Search Strategy. (DOC)

S1 Table Risk of Bias. (DOC)

S2 Table Baseline Characteristics—Intravenous vs Oral Rehydration Therapy. (DOC)

S3 Table Baseline Characteristics–Antiemetics. (DOC)

S4 Table Baseline Characteristics–Probiotics. (DOC)

S5 Table Baseline Characteristics—Intravenous Fluids. (DOC)

S6 Table PRISMA Checklist. (PDF)
