# Correction: Gastroenteritis Therapies in Developed Countries: Systematic Review and Meta-Analysis

**DOI:** 10.1371/journal.pone.0176741

**Published:** 2017-04-26

**Authors:** Stephen B. Freedman, Dion Pasichnyk, Karen J. L. Black, Eleanor Fitzpatrick, Serge Gouin, Andrea Milne, Lisa Hartling, Pediatric Emergency Research Canada Gastroenteritis Study Group

There is an error in the first sentence of the second paragraph under the subheading “Oral Rehydration Therapy (ORT)” in the Results section. The correct sentence is: Three studies provided data on the effect for the primary outcome of hospitalization, which in meta-analysis, showed no significant difference between groups (RR 1.51, 95% CI 0.75, 3.03, I^2^ = 11%; Table 4; Fig 2).

There are several errors in Fig 2. Please see the corrected Fig 2 here.

**Fig 2:**
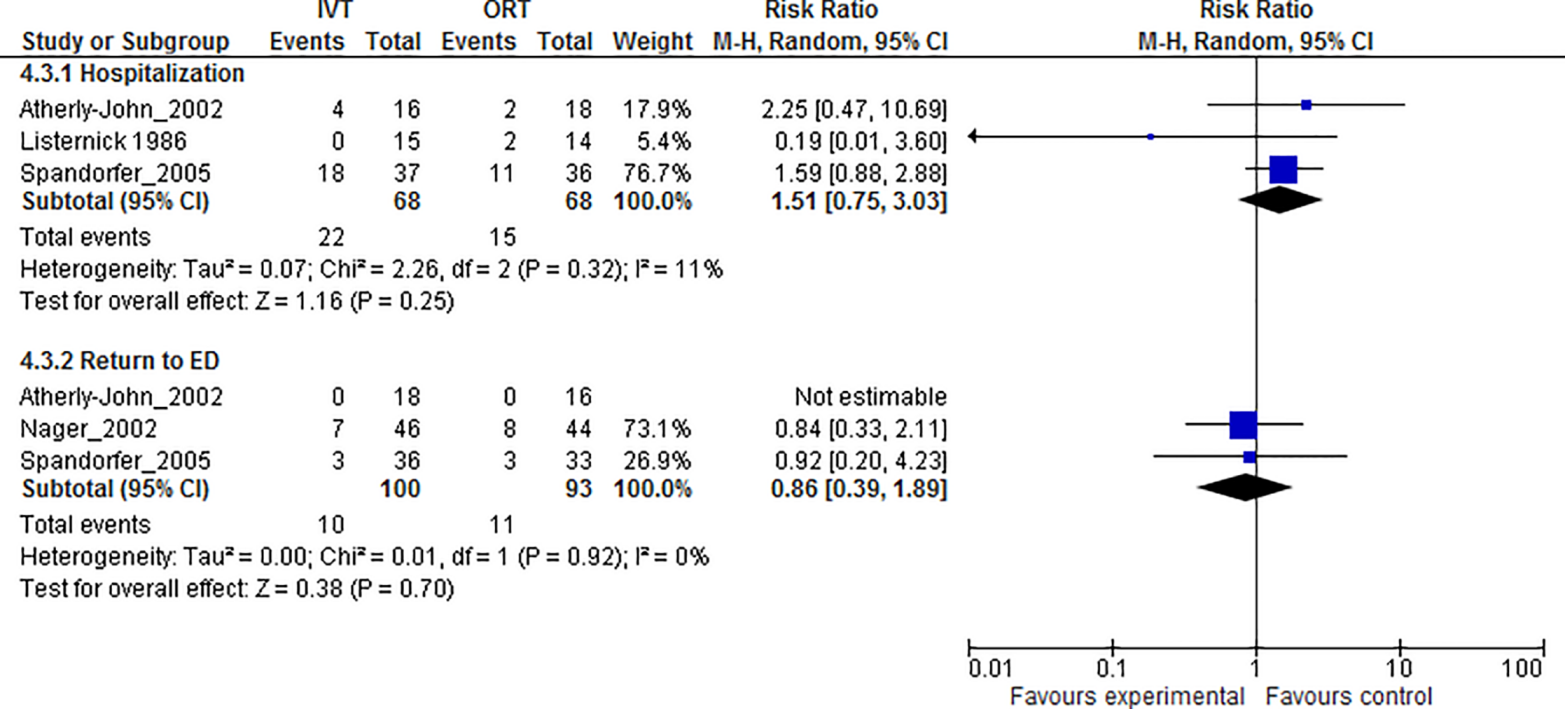
Meta-graph comparing oral rehydration therapy vs. intravenous fluid therapy. Results from meta-analysis of direct comparisons of oral rehydration therapy vs. intravenous fluid therapy on the outcomes of admission to hospital from the emergency department and revisits to the emergency departments, displayed employing Forest plots.
