# Supplementary material for: Gastroenteritis Therapies in Developed Countries: Systematic Review and Meta-Analysis
Source: PLoS One. 2015 Jun 15;10(6):e0128754. doi: 10.1371/journal.pone.0128754 (PMC4468143; doi:10.1371/journal.pone.0128754)
Supplement: S1 File — (PDF) [file pone.0128754.s001.pdf]

1. RESEARCH QUESTION

1.1. Objectives of the Proposed Project

The ***overall goal*** of this project is to synthesize the evidence on the management of acute gastroenteritis in children employing data specific to children in developed countries (i.e. the Canadian context). The results of this knowledge synthesis endeavour will provide an evidence base for the future development of a Canadian Consensus Statement. We will achieve our overall goal through the following ***specific objectives***:

1. To systematically and comprehensively identify and synthesize systematic reviews (SR) evaluating the efficacy of common interventions for the management of acute gastroenteritis
2. To update the searches for SRs identified in objective 1
3. To re-analyze and synthesize the data based on parameters relevant to developed countries
4. To conduct a SR of studies evaluating the diagnostic test accuracy of non-invasive methods of dehydration assessment

1.2. Knowledge-to-Practice Gap to be Filled

Gastroenteritis is an extremely common disease accounting for nearly 240,000 emergency department (ED) visits annually in Canadian children,[1] the hospitalization of 1 in 25 Canadian children by 5 years of age, and 13% of all childhood hospitalizations.[2] In 2001, the annual cost per 100,000 population in Canada was $11,465,541.[3] With the Canadian population nearing 34 million, we estimate that annual costs now exceed 4 billion dollars. Despite being a ubiquitous illness, there exists significant variation in the management of gastroenteritis around the world. In North America there is evidence of significant variation at institutional,[4] national,[5] and international (Canada/US) levels.[6] Our recently completed practice pattern survey of North American pediatric emergency medicine (PEM) physicians[6] and 11-centre, prospective, cross-Canada, practice pattern analysis[5] documented substantial variation across North America and within Canadian academic pediatric EDs. Moreover, the greatest source of variation appears to be the individual ED where the care is provided.[5] Such variation may stem from inconsistent implementation of clinical practice guidelines (CPG) or medical directives. In a survey we recently completed of Ontario's 162 EDs, we found that only 28% have a gastroenteritis CPG/pathway and 38% have an oral rehydration therapy medical directive (manuscript under review).

In Canada, there is no comprehensive guideline on the management of pediatric gastroenteritis. In 2006 the Canadian Paediatric Society (CPS) issued a position statement restricted to the use of oral rehydration therapy and refeeding.[7] In 2011 a position statement was published on the use of oral ondansetron.[8] The lack of a global management document clearly guiding therapy has resulted in substantial practice variation. Although a more comprehensive, but somewhat outdated (2003) document is endorsed by The American Academy of Pediatrics (AAP)[9] it contradicts the CPS's statement on antiemetics[8] and the European Society for Gastroenterology Hepatology and Nutrition's stance on probiotics.[10] While SRs have evaluated treatment options such as oral rehydration,[11] antiemetics,[12, 13] and probiotics,[14-18] they are inconclusive or discordant. Moreover, the applicability of existing SRs for use in **developed countries** is limited because they include a heterogeneous group of studies in terms of population and setting. This is a crucial point because there are two bodies of gastroenteritis research – studies performed in ***developing*** and ***developed*** nations. This distinction is critical when assessing generalizability as the etiologic agents and the nutritional status of the infected populations vary immensely as do the most pertinent outcomes. Studies from developing nations include more severe cases, organisms rarely seen in developed nations, and malnourished children who are at risk for chronic diarrhea and even death.[19, 20] In developed countries the focus is on minimizing morbidity (e.g. intravenous (IV) insertion and hospitalization). Thus, SRs performed to date cannot be generalized to the Canadian setting.[21] Moreover, the **reviews conducted to date have lacked relevance to parents and children** with most studies focusing on isolated symptoms (e.g. duration of

diarrhea[18] or vomiting[13]) instead of relevant outcomes (e.g. IV insertion, admission, revisits).[21] The lack of clinical relevance of some SRs has been identified regarding the management of acute asthma, with experts concluding that despite the existence of SRs, their usefulness is hampered by poorly defined populations, unimportant outcomes and the insufficient identification and handling of heterogeneity.[22] This problem is clearly present as it relates to gastroenteritis with an analysis of 138 pediatric gastroenteritis randomized clinical trials (RCT) identifying 64 unique definitions of diarrhea, and 69 of "resolution".[21] Lastly, previously performed SRs exist as stand-alone entities examining single interventions or groups of interventions (e.g. probiotics). While such SRs are highly effective in bringing together multiple trials, comparative data across interventions are lacking.

To address these shortcomings, we will conduct an overview and update of SRs using rigorous methods.[23] We will re-analyze the data specific to the context of developed countries. This will provide a comprehensive synthesis in one accessible and usable document of all relevant interventions for the management of gastroenteritis in children in developed countries. **Our synthesis will focus on well-defined populations, key interventions, and clinically important and well-defined outcomes** to minimize heterogeneity and maximize relevance for Canadian decision makers. The topics selected were identified by our research team and knowledge users based on **(1)** clinical importance, **(2)** recent publications, and **(3)** practice uncertainty. These **topics** are:

1.2.1. Oral Rehydration Therapy: The importance of this intervention is highlighted by position statements by the CPS,[7] the AAP[24] and a Cochrane Review published by the Co-Principal Applicant (PA).[11] Despite strong evidence in favor of the use of oral rehydration, there continues to be variation in clinical practice as evidenced by our reporting a 29% absolute difference in its routine usage between US and Canadian PEM physicians,[6] and variation in the usage of IV rehydration (i.e. failure or non-use of oral rehydration therapy) across Canada (6-66%).[5] Moreover, research regarding its use continues to emerge with an OVID search for "gastroenteritis" AND "oral rehydration therapy" limited to children in the past 5 years yielding 119 publications. Updating the previous SR with this more recent evidence and exploring results specific to developed countries (i.e. the Canadian context) would provide a more solid evidence base for informed and consistent practice.

1.2.2. Antiemetics: The use of antiemetics is highly controversial, variable, and of great importance. Although popular in Canada, dimenhydrinate is rarely used elsewhere and has been the topic of two recent studies (including one by a Co-A).[25-27] Ondansetron use is debated as well with a recent SR concluding that the evidence favouring ondansetron use is limited[13] while an earlier SR[12] and a CPS position statement[8] concluded that ondansetron use results in clinical benefits and it should be incorporated into guidelines. The AAP guideline states that "cost-effectiveness analyses should be undertaken."[9] The latter has been done by the Co-PA – its appropriate use would result in a savings of $1.7 million to Canadians annually.[1] The varying conclusions of these reviews has resulted in practice variation between countries (US-67%, Canada-45%)[6] and among Canadian pediatric EDs (0-38%).[5]

1.2.3. Probiotics: Interest and confusion regarding the use of probiotics is highlighted by recent SRs. A 2010 Cochrane Review which included 63 studies with 8,014 participants[18] concluded that "more research is needed to guide the use of particular probiotic regimens in specific patient groups."[18] The fact that only 15% of North American PEM physicians administer probiotics to children with gastroenteritis highlights the uncertainty regarding their use.[6] PEM physicians have indicated that they do not feel the evidence is sufficiently compelling to routinely recommend their use.[6] Further, the endpoints evaluated in the SRs to date (e.g. duration of diarrhea, number of stools on day 3) are not clinically relevant.[6] Consequently, current usage at discharge from Canadian EDs ranges from 0-17%.[5]

1.2.4. Intravenous (IV) Fluid Therapy: While IV fluid therapy is widely and frequently used, position statements provide little guidance on the optimal fluid administration rate and composition.

The current standard is to administer a 0.9% sodium chloride bolus followed by a hypotonic solution ranging from 0.2-0.45% sodium chloride to replace the remaining deficit plus maintenance.[28] This approach is questioned[29, 30] because of reports of death or permanent neurologic impairment from hyponatremic encephalopathy.[31-33] Emerging evidence indicates that the incidence of hospital-acquired hyponatremia is significant in children with dehydration from gastroenteritis.[34, 35] Experts argue that a safer and more effective approach would be to administer 0.9% sodium chloride following bolus therapy.[28] This controversy has resulted in significant variations in the administration of IV fluid therapy.[5] The latest SR (2007) concluded that further evidence is required to reach a firm conclusion.[36]

1.2.5. Non-Invasive Dehydration Assessment: Dehydration severity is the cornerstone of gastroenteritis management. All published guidelines recommend that therapy be instituted based on dehydration severity.[9, 10] An example of the role played by dehydration assessment is depicted in The Hospital for Sick Children's CPG (Appendix Figure 1). At present, the "gold standard" method of assessing dehydration severity is the change in body weight.[37] Unfortunately, recent well weights are rarely available.[38] Thus, research has focused on clinical scores employing a combination of signs and symptoms[39-42] and recently on emergency bedside ultrasound.[38, 43, 44] Prior to their development, a SR concluded that the inaccuracy of available tests limits the ability of clinicians to estimate the exact degree of dehydration.[45] Given the importance of this topic and recent publications, knowledge synthesis is needed to resolve practice uncertainty. More importantly, with bedside ultrasound becoming a standard tool in EDs across North America, its role in dehydration assessment needs to be more clearly defined.[46] By enabling clinicians to use the most appropriate method of dehydration assessment, subsequent therapeutic options (Sections 1.2.1 – 1.2.4) can be appropriately selected.

1.3. Description of and Relevance to the Identified Knowledge Users

This proposal brings together leaders in gastroenteritis research (SF, DJ, SG, KB), evidence synthesis (LH), and key organizations in order to clarify outstanding questions in managing gastroenteritis. Four organizations are represented by knowledge users: the CPS (BH), the Canadian Association of Emergency Physicians (CAEP; FB), the Pediatric Emergency Administrators for Children's Hospitals (PEACH; MJ), and TRanslating Emergency Knowledge for Kids (TREKK; TK). These organizations are directly involved in decisions that affect institutional policies, physician practices, and the care that children receive. Our proposal is directly in line with their missions. The CPS's mandate is to advance the health of children via continued professional development through position statements, a peer-reviewed journal, and educational opportunities. CAEP's mission is to advocate for patients requiring emergency medical care, to be a leader in emergency medicine education, and to provide a forum for research. PEACH is comprised of the medical and nursing directors of Canada's children's hospitals. Their goal is to set the national standard of pediatric emergency care through the sharing of data, innovations, best practices, and protocols. TREKK's mission is to actively engage in knowledge mobilization to minimize the evidence-practice gap. Our partnership with the TREKK Network is unique as the Co-PAs are integrally involved in this network. LH is a Co-Director responsible for knowledge synthesis, while SF is the Ontario Region's Nodal leader. Moreover, our knowledge synthesis topic has been targeted by TREKK's leadership as a priority knowledge mobilization topic.

1.4. How does the Project respond to the Funding Opportunity

The goals of this proposal are directly aligned with the objectives of this funding opportunity: to increase the application of knowledge in decision-making by producing a synthesis that responds to the information needs of knowledge users, and to extend the benefits of knowledge synthesis to new kinds of questions relevant to knowledge users that have not traditionally been synthesized. Additionally, the objectives are directly in step with those of the CIHR-Institute of Human Development, Child and Youth Health to promote and support research that improves the health of infants and children.

2. RESEARCH APPROACH

2.1. Research Methods

Our approach will involve four **key steps**. We will: **(1)** conduct an overview of reviews to identify all relevant SRs performed to date**; (2)** search for new evidence to update existing SRs; **(3)** re-examine the evidence relevant to the Canadian setting (i.e. focus on studies that examine clinically relevant outcomes, in outpatients, in developed countries); and **(4)** conduct a SR to synthesize evidence to determine the diagnostic characteristics of non-invasive methods of dehydration assessment.
Our approach involves the basic methodology advocated by The Cochrane Collaboration[23] enhanced by the most current methodological evidence. We will follow standard, documented procedures for refining the research questions, searching the literature, screening and selecting studies for inclusion, assessing methodological quality and bias, synthesizing results, meta-analyzing data, grading the evidence, interpreting results, and disseminating findings to knowledge users. We will adhere to several **guiding principles** by: **(1)** using a multidisciplinary approach, (i.e. methodologists, statisticians, librarians, content experts, and knowledge users); **(2)** working to develop focused, answerable questions and develop a priori protocols in collaboration with technical and content experts, and key stakeholders; and, **(3)** employing methods that minimize bias in the conduct and reporting of our research. These methods will ensure that our work is relevant, transparent, and reproducible.

2.1.1. Overview of Systematic Reviews (Objective 1)

Objective: To systematically and comprehensively identify and synthesize SRs evaluating the efficacy of the following common interventions for the management of acute gastroenteritis: **(1)** oral rehydration therapy; **(2)** antiemetics; **(3)** probiotics; and **(4)** IV fluid therapy.
Search Methods: A medical librarian with experience in searching for SRs will develop a search strategy in collaboration with the content experts. We will use previous SRs and work done by the co-applicants as a starting point for identifying relevant key words. We will: **(1)** systematically search MEDLINE, EMBASE, the Cochrane Library, and for grey literature; **(2)** hand-search appropriate journals and major, relevant scientific meetings (e.g., Society for Pediatric Research); **(3)** check reference lists of relevant reviews; and **(4)** contact primary authors. The search will not be restricted by language or publication status but will be restricted to 2000 onwards in order to identify the most recent SRs. We will maintain detailed documentation of search strategies, dates, and results.
Study Selection: Search results will be screened independently by two reviewers to identify potentially relevant citations. The full text of all potentially relevant citations will be obtained and assessed for inclusion using a standard form with predefined eligibility criteria. Disagreements will be resolved by consensus. Decisions regarding inclusion and reasons for exclusion will be documented.
Inclusion Criteria: Studies will be included based on the following criteria:
Design: SRs, defined as reports that meet the following criteria:[23] clearly stated objectives with pre-defined eligibility criteria; explicit, reproducible methodology; systematic search strategy to identify all studies meeting eligibility criteria; assessment of study validity; systematic presentation and synthesis of the characteristics and findings of the included studies.
Population: Children <18 years of age with acute gastroenteritis. Reviews that include studies with individuals ≥18 years of age will be included if data are presented separately for children.
Interventions: (1) any oral rehydration therapy vs. IV or nasogastric rehydration; (2) any antiemetic vs. placebo or another intervention; (3) any probiotic vs. placebo or another intervention; and (4) different rates and compositions of IV fluids.
Data extraction: One reviewer will extract data from the reviews using a structured form that captures: characteristics of the review; participants; interventions; outcomes; funding source; results; and, conclusions. Data extraction will be verified by a second reviewer for accuracy and completeness. This approach is supported by previous research we have conducted.[47]

Assessment of methodological quality: Two reviewers will independently assess the quality of SRs using AMSTAR (Appendix, Table 1).[48] Discrepancies will be resolved by discussion or third person.
Data synthesis: We will use a qualitative approach to synthesize the evidence. We will describe the SRs and develop evidence tables according to: objectives of the SR; the Population Intervention Comparison Outcome (PICO) elements; study designs included; search dates; methodological quality; and, overall conclusions. The intent will be to map the existing SRs to identify where updating is required and which SRs provide evidence for further analyses specific to developing countries.

2.1.2. Updating the Searches of Systematic Reviews (Objective 2)

Objective: To re-run the searches for the SRs identified in objective 1.
Search Methods: A medical research librarian will develop comprehensive search strategies for the key interventions: **(1)** oral rehydration therapy, **(2)** antiemetics, **(3)** probiotics, and **(4)** IV fluid therapy. We will search the Cochrane Central Register of Controlled Trials, MEDLINE, EMBASE, and the grey literature; hand-search relevant conferences; screen reference lists and contact primary authors of relevant studies. Search dates will correspond to the start dates of the aforementioned electronic libraries as some non-Cochrane SRs only search MEDLINE or do not employ comprehensive search strategies. Thus, important primary studies may not have been identified in the original SR search.
Study Selection: The same process will be followed as previously described (Section 2.1.1).
Inclusion Criteria: We will include only RCTs.[49] The population and interventions are the same as previously described (Section 2.1.1).
Data Extraction and Synthesis: We will identify new studies that add to the evidence base for the comparisons of interest. New studies will be considered for further analysis (see Section 2.1.3).

2.1.3. Analysis and Synthesis based on the Canadian Context (i.e. developed countries) (Objective 3)

Objective: To re-analyze the data based on parameters relevant to Canadian stakeholders.
Study Selection: Using methods described above, we will identify relevant studies from the included SRs and the updated searches.
Inclusion Criteria:
Design: RCTs.[49]
Population: Studies will be included if they were conducted in EDs or similar outpatient settings in developed nations - Australia, Canada, Europe, Japan, New Zealand, and the United States.[50]
Interventions: As above for objective 1.
Outcomes: Outcomes will vary based on the intervention being evaluated as a single outcome cannot be applied to all interventions. They are each anticipated to have an impact on different events that may occur during an episode of acute gastroenteritis (Appendix, Table 2). A common theme - **healthcare resource utilization as a measure of the effectiveness of the intervention** - will take priority.[51] The selected outcomes are based on recommendations to employ outcomes of interest to parents, clinicians, and health systems. For example, the US Agency for Healthcare Research and Quality has listed the "Pediatric Gastroenteritis Admission Rate" as a Quality Indicator that provides information on the quality of hospital care and the adequacy of local outpatient and other health services.[51] The following outcomes will enable us to identify and promote the best practices in healthcare – a strategy identified in CIHR's "Strategy for Patient-Oriented Research"[52]: **oral rehydration** (primary: admission rate; secondary: length of stay [LOS], return visits, adverse effects [AEs]); **antiemetic agents** (primary: administration of IV rehydration; secondary: admissions, LOS, ED return visits, AEs); **probiotic agents** (primary: subsequent healthcare provider visits within 1 week; secondary: administration of IV fluids, AEs); **IV fluid therapy** (primary: LOS; secondary: admissions, ED return visits, dysnatremias).
Assessment of included studies for risk of bias: The Cochrane Risk of Bias (RoB) tool[23] will be used to assess the internal validity of RCTs based on six domains (Appendix, Table 3). Each study will be

assessed independently by two reviewers (one with methodological expertise, the other with content expertise). Disagreements will be resolved by consensus, or involving a third reviewer as required.

Data extraction: The same process for data extraction will be followed as for objective 1 and will include the following items: study characteristics (e.g., date of publication, clinical setting, country), participants (e.g. age, gender), interventions and comparisons (e.g. type, dose, route, timing, duration), outcomes (e.g. types, timing), use of intention-to-treat protocols, funding, and results.

Data synthesis: Data synthesis will be done within the different interventions/comparisons of interest. First, we will ***qualitatively*** describe the study populations, settings, interventions, and outcome measures. This will alert us to the possibility and sources of heterogeneity. Evidence tables will be developed describing the studies which will include information on: design, setting, sample size, protocol, treatment comparisons, methodological strength/risk of bias, outcomes, and results with effect sizes. We will conduct a ***quantitative*** analysis to numerically summarize the effectiveness of each intervention and to investigate sources of heterogeneity. We will report data for continuous outcomes as mean differences; these will be combined, where appropriate, using either a weighted mean difference or standardized mean difference and inverse-variance methods.[53] We will report data for dichotomous measures using relative risks, odds ratios, or risk differences. These will be combined using the most appropriate method given the data.[53] The primary analysis will be based on a random effects model.[54] We will perform ***indirect comparisons*** where little direct evidence is available comparing two interventions, but information is available comparing each intervention to a third intervention.[55] We will quantify ***heterogeneity*** using the I-squared statistic[56, 57] and classify it for each outcome as negligible, minimal, moderate, or substantial. We will explore reasons for heterogeneity including: statistical, methodological, and clinical. We will conduct ***subgroup analyses*** for children aged 3 months to 5 years as younger children are more prone to requiring therapeutic interventions. We will conduct sensitivity analyses based on fixed effects model, overall risk of bias, intention-to-treat analysis, and funding source. We will test for ***publication bias***, when sample size is sufficiently large, by means of a funnel plot,[58] and the rank correlation test,[59] weighted regression,[60] and the trim and fill method.[61] Analyses will be conducted using Review Manager 5.0, Stata 7.0, and S-Plus 7.0.

Grading the evidence: We will grade the quality of the available evidence using methods developed by the GRADE Working Group.[62] For each comparison and outcome specified above, we will assess the following domains: risk of bias, consistency, directness, precision, and publication bias. Overall quality of evidence will be graded as high, moderate, low, or very low. Decision rules will be developed a priori. Two reviewers, one with methodological expertise, the other with clinical expertise, will grade the evidence and resolve discrepancies through discussion or with third party input. This step is critical as a basis for informed decision making.

2.1.4. Systematic Review of Methods of Clinical Dehydration Assessment (Objective 4)

Objective 4: To conduct a SR evaluating the diagnostic test accuracy of two methods of clinical dehydration assessment: **(1)** clinical dehydration scores; **(2)** emergency bedside ultrasound.

Search methods: The methodology will be similar to that described above (Section 2.1.1).

Study selection: The methodology will be similar to that described above (Section 2.1.1).

Inclusion criteria:

<u>Design:</u> RCTs and prospective cohort studies.

<u>Population:</u> Studies will be included if they: **(1)** were conducted in EDs or similar outpatient settings; **(2)** were in developed nations; and **(3)** included participants <18 years of age with acute gastroenteritis. Studies with individuals ≥18 years of age will be included if data are available separately for children.

<u>Interventions:</u> Studies should compare a dehydration assessment to an objective measure of dehydration severity (e.g. difference between convalescent weight and ill weight).

<u>Outcomes:</u> Our primary outcome will be the ability of the diagnostic test to determine the severity of dehydration as both a continuous and categorical variable (i.e. none, some, severe).[9]

Assessment of included studies for methodological quality: Quality will be assessed using the QUADAS tool[63] as recommended by The Cochrane Collaboration.[64] This is a standard tool used in diagnostic SRs that assesses the representativeness of the study population, the verification procedure, blinding of test interpretation, and missing data. Two reviewers will independently apply the tool and resolve disagreements through consensus or with input from a third reviewer.

Data extraction: We will employ the same process as described above (Section 2.1.1) to extract the following information: design features, population characteristics, comparisons, and results.

Data synthesis: We will follow methods recommended by The Cochrane Collaboration for analysis and presentation of data for SRs of diagnostic test accuracy.[65] We will synthesize data separately for the different methods of dehydration assessment and reference standards used across studies. We will calculate sensitivity and specificity and combine results across studies that have sufficient clinical, methodological, and statistical homogeneity. We will produce summary ROC curves pending availability and homogeneity of data. Analyses will use a random effects model and the software described above. Meta-regression will be used to investigate whether test accuracy varies by study population, disease prevalence, test threshold, and methodological quality.

2.2. Knowledge User Team Member Engagement

Early on we identified four knowledge users who play key roles within organizations at the centre of decision making regarding pediatric health policies, programs and practice. **Dr. Bob Hilliard** is the Past-President of the CPS, and Former Chair of the Paediatric Specialty Committee of the Royal College of Physicians and Surgeons of Canada (RCPSC). He served as the chair of the CPS' Annual Conference for 6 years and is well aware of the needs of academic and non-academic clinicians. **Dr. Francois Belanger** has extensive experience as a practitioner, policy/decision-maker and healthcare administrator. He has worked in many different roles across Canada (e.g., Medical Director of the Montreal Children's Hospital; Chair, Pediatric Emergency Specialty Committee of the RCPSC; Division Head, Pediatric Emergency Medicine, Calgary Health Region). He is the Past-President of CAEP, and is currently the Acting Executive Vice-President and Chief Medical Officer for Alberta Health Services. In this role he is responsible for engaging Alberta's 7,200 physicians and advising the CEO and Board on issues related to clinical care, policy, and resource utilization. **Dr. Mona Jabbour** is Chair of PEACH. This link to the directors at each of the country's pediatric EDs will enable us to disseminate the results widely, thereby influencing institutional policies and the practices of individual emergency physicians. **Dr. Terry Klassen** is a member of the Institute of Medicine, and is the Research Director at the Manitoba Institute of Child Health. He has founded numerous collaborative networks including TREKK, the Alberta Research Centre for Health Evidence (ARCHE), Pediatric Emergency Research Canada (PERC), and the Cochrane Child Health Field. He is currently the global leader of Pediatric Emergency Research Networks (PERN) and was the founding Co-Editor-in-Chief, Evidence Based Child Health, and the Co-Creator of the StaR Child Health Network.

The knowledge user Co-Applicants will be involved in both integrated and end-of-grant knowledge translation (KT) by: **(1)** contributing to the focus of the research questions and analysis, **(2)** being involved in data collection and tool development, and **(3)** assisting with development of the SRs and eventual dissemination of findings (i.e. KT into CPG and uptake into practice). Specifically, *the knowledge users will participate in*: **(1)** refining and approving the research questions at the outset of the project; **(2)** identifying potentially relevant studies; **(3)** reviewing interim results; **(4)** suggesting additional analyses; **(5)** interpreting results; and **(6)** assessing the applicability of the results for their knowledge user groups. Regarding *dissemination*, the knowledge users will assist with knowledge transfer activities and dissemination of the results among their stakeholder groups. The attached letters of support indicate their commitment to this initiative and willingness to be involved.

2.3. Interactive Sessions

We will use **integrated KT** (I-KT) methods throughout the project to ensure that knowledge users are involved in the research process itself. Researchers and knowledge users will work together to identify the crucial research questions and to decide on methodology. Following the conduct of the SRs, the research team will work together to interpret and disseminate the findings. Employing an I-KT approach will ensure the production of research results that are highly relevant and likely to be used by knowledge users to improve health and the health system. To optimize our I-KT plan we will employ: **(1)** monthly email updates; **(2)** bi-monthly conference calls; and **(3)** interactive sessions between the co-applicants and knowledge users. The latter will include a ***teleconference*** once the preliminary analyses have been conducted and an interim report has been circulated. This teleconference will be held to: **(1)** review the preliminary results; **(2)** identify additional analyses that would be informative; **(3)** provide context for interpretation of the results; and **(4)** identify methods for early and rapid dissemination of the findings (e.g. posting outcomes on websites such as the CPS, CAEP, and TREKK). An ***in-person meeting*** will be held following the production of the final SR manuscripts to: **(1)** review the findings and conclusions; and **(2)** assess the appropriateness of applying for a subsequent CIHR ***planning grant*** to identify research questions and priorities that could form the basis of a grant application. This teleconference will enable an evaluation of the state of the evidence and **(3)** the appropriateness (including concept and leadership) of applying for a CIHR ***dissemination event*** grant to enable: KT strategies that will educate patients, health professionals, community organizations, policy-makers, and the general public regarding best practices including clinical care, policy and decision making; and **(4)** evaluate the appropriateness (including concept and leadership) of applying for a CIHR ***knowledge translation supplement*** grant to enable: the development of websites, plain language summaries and knowledge exchange tools (e.g. educational CD-ROMs, decision support tools); the production and distribution of written materials in various formats; the dissemination of research results through specialized publications; and meetings/presentations/webinars (i.e. linkage and exchange activities) and Café Scientifique to implement a broader KT strategy. These activities will be done in collaboration with TREKK whose mandate is knowledge mobilization to both professional and consumer audiences.

2.4. End-of-Grant Knowledge Translation Plan

Our immediate dissemination strategy will target the clinicians, medical directors, and administrators who are responsible for the care of children with gastroenteritis. The key deliverable that will stem from this initiative is an **evidence synthesis**, including several SRs, which will improve the overall health of children while simultaneously containing health care costs. Intangible benefits will include reassurance to parents that children are receiving optimal treatment while avoiding unnecessary IV hydration and hospitalization. Finally, by reviewing all currently available evidence in the field, we will be able to identify evidence gaps for future research. We will prepare manuscripts, according to established reporting guidelines[66, 67] for publication in peer-reviewed journals. Several manuscripts are likely to emerge from the extensive evidence synthesis that is planned. We have secured interest in these publications from a variety of sources including: **(1)** *Paediatrics and Child Health* (the journal of the CPS) – a user-friendly summary of the evidence synthesis, with a focus on implications for practice and research will be published in the journal. This summary is a regular contribution by ARCHE which employs a clinical question and then provides a response and clinical commentary based on the evidence. **(2)** *Evidence-based Child Health: A Cochrane Review Journal* (produced by ARCHE) – will include the results of the evidence synthesis work in addition to a specific piece on the overview of SRs. **(3)** Preliminary discussions have also taken place with Dr. Lewis First (Editor-in-Chief, *Pediatrics*) who expressed interest in publishing our topic as a State-of-the-Art Review and Dr. Sharon Straus (Associate Editor, Guidelines, *CMAJ*) who suggested that the evidence synthesis could be

submitted as an original research paper. The findings will also be submitted for presentation at the annual meetings of The Pediatric Academic Society, the CPS, the CAEP, and the Society for Academic Emergency Medicine.

Following the publication of the SRs, they will be posted on the websites of the partner knowledge user organizations (i.e. CPS, CAEP, PERC, TREKK). Further, it is our intent to enhance our end-of-grant KT plan by applying for supplemental funding as described above (Section 2.3). It is our intent to use these supplemental funds to hold a conference, modeled after the structure developed by the Consensus Development Program of the US National Institutes of Health (NIH),[68] to develop a ***CPG*** for dissemination through the Pediatric Emergency Sections of the CPS and CAEP. Once developed, it will be presented at the annual multidisciplinary PERC conference which brings together Canada's 14 children's hospitals.

Co-applicants (DJ, LH) and knowledge user (TK) were crucial in obtaining a CIHR Team Grant in PEM which has enabled the development of an electronic network that will be used to disseminate the results to over 350 PEM providers. These individuals are heavily involved in training PEM fellows; pediatric, emergency medicine and family medicine residents; and a large number of medical students, advanced practice nurses, and physician assistants. Moreover, as a member of the Steering Committee of the Pediatric Emergency Medicine Collaborative Research Committee of the Section of Emergency Medicine of the AAP, the Co-PA (SF) is well positioned to disseminate the results to relevant US stakeholders.

This initiative ties directly into the objectives of the ***TREKK Network*** which has recently been awarded funding (Appendix, Attachment 1) through the National Centres of Excellence Knowledge Mobilization program. The director of the network (TK) is a knowledge user on this grant and three of the TREKK co-directors are also co-applicants or knowledge users (LH, DJ, and MJ) (Appendix, Figure 2 TREKK Organizational Chart). The network will partner 13 academic institutions with 31 community hospitals with the goal of enhancing knowledge mobilization. Central to the proposal are **(1)** the conduct of interviews and focus groups with the network's medical and operations directors to identify barriers to dissemination, implementation, and uptake of CPGs. This information will **(2)** inform the development and evaluation through a cluster RCT of KT strategies designed to enhance the uptake of our gastroenteritis CPG which will be based on the products of our knowledge synthesis grant. With the assistance of the TREKK Knowledge Broker, we will develop and disseminate the results of our work as well as our planned ***knowledge translation supplement*** grant.

The **clinician Co-Applicants** will ensure that the knowledge generated by this grant responds to the needs of the true end-users, i.e. clinicians who treat children. Both pediatricians (e.g. CPS) and non-pediatrician (e.g. CAEP, TREKK) health care providers will be targeted directly by our I-KT and end-of-grant KT strategies. The Co-PA (SF) will: **(1)** conduct interactive seminars at the annual conferences of three relevant organizations while the study is ongoing (CPS, CAEP, PERC). These will serve to explain the importance of the knowledge to be generated, and identify the needs of the end-users and the most appropriate methods of presenting results and disseminating findings; **(2)** conduct interactive seminars at the annual conferences of the same organizations the year after the study is completed to disseminate the results and discuss ongoing KT efforts; and **(3)** meet with the relevant sections and committees of these organizations (CPS - Acute Care, Community Pediatrics, Nutrition and Gastroenterology, Emergency Pediatrics; CAEP – Pediatric Emergency Physicians, and the Standards Committee). The ties to these organizations and the history of collaboration by the applicants and the knowledge users will ensure the successful dissemination of the recommendations that stem from this grant, and ultimately the uptake and implementation of effective strategies to manage children with acute gastroenteritis across Canada.

3. FEASIBILITY

3.1. Strengths of the Team

We have gathered an outstanding team with methodological expertise in SRs and clinical expertise in acute gastroenteritis and PEM. As the director of ARCHE, the Co-PA (LH) brings substantial experience in conducting SRs for a wide range of research questions, as well as conducting methodological research to improve the way SRs are done.[47, 69-77] LH was Co-PA on a CIHR Knowledge Synthesis Grant funded in 2008; the results of this project were published in the *British Medical Journal* in April 2011. The project provided evidence for the comparative effectiveness of different treatments for bronchiolitis and involved novel, cutting-edge, statistical methods (i.e. mixed treatment comparisons). ARCHE has staff in place to carry out the work proposed including individuals with extensive experience in project management, information sciences, meta-analysis, and medical writing and editing. The team also includes internationally-renowned researchers (particularly the Co-PA, SF) in acute gastroenteritis who have a long history of collaboration. Together these individuals have conducted a number of studies[1, 5, 78-85] and meta-analyses[11, 86] on the management of gastroenteritis. The applicants have recently completed major RCTs evaluating IV rehydration methods (SF-BMJ; in press – Appendix, Attachment 2)[85] and the use of an antiemetic (SG-presented at several conferences in the spring/summer of 2011).[27] Additionally, the Co-PA (SF) has recently completed an evaluation of a clinical dehydration score (manuscript under review), has been involved in studying practice pattern variation in the treatment of acute gastroenteritis in pediatric EDs across Canada and North America,[5, 6] and has previously developed a CPG for acute gastroenteritis.[87] Hence, team members have a significant amount of unpublished, yet crucial data.

3.2. Contributions to the Project (Appendix, Table 4)

The Co-PA (LH) will oversee all aspects of the synthesis work, including the overview of reviews, updating reviews, and the SR of non-invasive methods of dehydration assessment. She will: **(1)** supervise staff at ARCHE in the conduct of each step, **(2)** be involved in piloting all forms, **(3)** perform quality and grading assessments as the methodological reviewer, and **(4)** guide the analysis and lead the writing, along with the Co-PA (SF), of publications and other dissemination products. The Co-PA (SF) will: **(1)** be involved in all stages of the review process, **(2)** provide direction with respect to the clinical context and relevant clinical decisions, **(3)** be involved in screening and inclusion, quality assessment, grading evidence, data extraction, **(4)** guide the analysis, and **(5)** oversee and participate in all knowledge dissemination activities. The co-applicants will provide clinical input at all stages of the review including focusing the research questions, specifying inclusion criteria, guiding the analysis, interpreting the results, and contributing to the preparation of dissemination materials. KB, EF, and SG will participate in screening studies for inclusion, quality assessments, and data extraction. DJ has extensive experience developing CPGs; hence, his perspective will be critical to aligning the research questions/synthesis activities with the ultimate end product. The contributions of the knowledge users have been outlined in Section 2.3, and individual responsibilities are listed in Appendix, Table 4. The Co-PAs and co-applicants will meet bi-monthly by teleconference and more frequently as needed. Ongoing, regular communication will occur via monthly email updates. ARCHE will also assign a project coordinator, research assistants, a librarian, and a statistician to the project.

3.3. Timeline

A detailed timeline is presented in Appendix, Table 5. We will undertake all proposed synthesis activities within 12 months, with some of the dissemination activities occurring thereafter. Time estimates are based on previous extensive experience conducting SRs at ARCHE. We have staggered synthesis activities to enable staff to be working on the different stages consecutively. Following completion of the preliminary analysis, we will hold an interim meeting with all co-applicants and knowledge users.

4. OUTCOMES

4.1. Project Impact

The results of this project will clarify the appropriate treatment for children with acute gastroenteritis and will have a significant impact at both patient care and healthcare system levels. Our evidence synthesis has the potential to result in numerous benefits including: **(1)** more consistent and appropriate management of children with acute gastroenteritis across Canada based on the most recent and rigorous evidence; **(2)** enhanced patient outcomes; **(3)** reduced inappropriate healthcare utilization; and ultimately, **(4)** reduced costs to the healthcare system.

Health outcomes: Over 10 years ago, a single pediatric institution demonstrated that the implementation of an evidence-based CPG could result in significant improvements in the health of children with acute gastroenteritis.[88] Following implementation, mean yearly admissions decreased by 33% and LOS decreased by 21% for children with minor illness. Total inpatient days/year decreased by 43%. Nonetheless, adherence to guidelines has remained poor and within the past 2 years, researchers have reported evidence greater guideline adherence at higher volume hospitals[89] and substantial variation in resource use, with individual hospitals contributing to the variance of mean LOS, total adjusted charges, and use of diagnostic studies.[90] Moreover, in our recent survey of Ontario EDs, we demonstrated that the majority lack evidence based gastroenteritis treatment guidelines. Thus, adherence to the best available evidence can improve outcomes, thus there is a need to synthesize and disseminate this knowledge to the end-users.

Practice: We and others have shown that the individual institution contributes significantly to practice variation even after adjustment for co-variates.[5, 90] By synthesizing the evidence relevant for developed countries with widespread endorsement from key stakeholder groups, we intend to reduce practice pattern variation thereby reducing the number of children receiving unnecessary IV fluids and hospitalization. Such interventions place children at unnecessary risk of iatrogenic injury,[34] and therefore should be avoided whenever not truly medically warranted. Our planned analysis of IV rehydration will enable us to provide guidance to reduce the risk of adverse events. Together, these benefits will lead to a reduction in healthcare costs through optimizing the management of children with gastroenteritis.[88, 90]

Programs and Policies: By having the support of key knowledge users (e.g. Past-President of the CPS, Director of TREKK, Past-President of CAEP, Chair of PEACH) we are well positioned to have the findings of this SR ready for translation into national programs and policies in a timely fashion. By bringing together researchers and knowledge users we will also be able to identify key areas where the evidence is lacking to target future research initiatives.

4.2. Project Relevance

This review will serve as a template for the treatment of other pediatric disease processes where there exists significant variation in the treatment provided. Even within individual EDs staffed by PEM physicians, overall practice variation can be significant[91] - 8-fold variation in hospital admission rates, 3-fold variation in IV therapy, and 5-fold variation in head computed tomography use. This results in longer than expected length of stay for those with higher than expected use of laboratory tests and imaging.[91] Moreover, return rates have not been found to be significantly correlated with resource use. Specific diseases where Canadian data describing significant practice pattern variation exists include the ED treatment of children with asthma,[92] bronchiolitis,[93] fevers,[94] fractures,[95] and migraines.[96] The activities and collaborations described herein provide an excellent model to synthesize evidence, engage end-users, and translate knowledge into practice to ensure that children receive optimal care with the most efficient use of healthcare resources.

1. Freedman SB, Steiner MJ, Chan KJ. Oral ondansetron administration in emergency departments to children with gastroenteritis: an economic analysis. PLoS Med 2010;7.
2. Malek MA, Curns AT, Holman RC, et al. Diarrhea- and rotavirus-associated hospitalizations among children less than 5 years of age: United States, 1997 and 2000. Pediatrics 2006;117:1887-92.
3. Majowicz SE, McNab WB, Sockett P, et al. Burden and cost of gastroenteritis in a Canadian community. J Food Prot 2006;69:651-9.
4. Powell EC, Hampers LC. Physician variation in test ordering in the management of gastroenteritis in children. Arch Pediatr Adolesc Med 2003;157:978-83.
5. Freedman SB, Gouin S, Bhatt M, et al. Prospective assessment of practice pattern variations in the treatment of pediatric gastroenteritis. Pediatrics 2011;127:e287-95.
6. Freedman SB, Sivabalasundaram V, Bohn V, Powell EC, Johnson DW, Boutis K. The treatment of pediatric gastroenteritis: a comparative analysis of pediatric emergency physicians' practice patterns. Acad Emerg Med 2011;18:38-45.
7. Oral rehydration therapy and early refeeding in the management of childhood gastroenteritis. Paediatrics and Child Health 2006;8:527-31.
8. Cheng A, Canadian Paediatric Society - Acute Care Committee. Emergency department use of oral ondansetron for acute gastroenteritis-related vomtiing in infants and children. Paediatr Child Health 2011;16:177-9.
9. King CK, Glass R, Bresee JS, Duggan C. Managing acute gastroenteritis among children: oral rehydration, maintenance, and nutritional therapy. MMWR Recomm Rep 2003;52:1-16.
10. Guarino A, Albano F, Ashkenazi S, et al. European Society for Paediatric Gastroenterology, Hepatology, and Nutrition/European Society for Paediatric Infectious Diseases evidence-based guidelines for the management of acute gastroenteritis in children in Europe. J Pediatr Gastroenterol Nutr 2008;46 Suppl 2:S81-122.
11. Hartling L, Bellemare S, Wiebe N, Russell K, Klassen TP, Craig W. Oral versus intravenous rehydration for treating dehydration due to gastroenteritis in children. Cochrane Database Syst Rev 2006;3:CD004390.
12. DeCamp LR, Byerley JS, Doshi N, Steiner MJ. Use of antiemetic agents in acute gastroenteritis: a systematic review and meta-analysis. Arch Pediatr Adolesc Med 2008;162:858-65.
13. Alhashimi D, Al-Hashimi H, Fedorowicz Z. Antiemetics for reducing vomiting related to acute gastroenteritis in children and adolescents. Cochrane Database Syst Rev 2009:CD005506.
14. Szajewska H, Mrukowicz JZ. Probiotics in the treatment and prevention of acute infectious diarrhea in infants and children: a systematic review of published randomized, double-blind, placebo-controlled trials. J Pediatr Gastroenterol Nutr 2001;33 Suppl 2:S17-25.
15. Huang JS, Bousvaros A, Lee JW, Diaz A, Davidson EJ. Efficacy of probiotic use in acute diarrhea in children: a meta-analysis. Dig Dis Sci 2002;47:2625-34.
16. Van Niel CW, Feudtner C, Garrison MM, Christakis DA. Lactobacillus therapy for acute infectious diarrhea in children: a meta-analysis. Pediatrics 2002;109:678-84.
17. Allen SJ, Okoko B, Martinez E, Gregorio G, Dans LF. Probiotics for treating infectious diarrhoea. Cochrane Database Syst Rev 2004:CD003048.
18. Allen SJ, Martinez EG, Gregorio GV, Dans LF. Probiotics for treating acute infectious diarrhoea. Cochrane Database Syst Rev 2010;11:CD003048.
19. Opintan JA, Newman MJ, Ayeh-Kumi PF, et al. Pediatric diarrhea in southern Ghana: etiology and association with intestinal inflammation and malnutrition. Am J Trop Med Hyg 2010;83:936-43.
20. Al Jarousha AM, El Jarou MA, El Qouqa IA. Bacterial enteropathogens and risk factors associated with childhood diarrhea. Indian J Pediatr 2011;78:165-70.
21. Johnston BC, Shamseer L, da Costa BR, Tsuyuki RT, Vohra S. Measurement issues in trials of pediatric acute diarrheal diseases: a systematic review. Pediatrics 2010;126:e222-31.
22. Boluyt N, van der Lee JH, Moyer VA, Brand PL, Offringa M. State of the evidence on acute asthma management in children: a critical appraisal of systematic reviews. Pediatrics 2007;120:1334-43.

23. Higgins JPT, Green S. Cochrane Handbook for Systematic Reviews of Interventions Version 5.0.2 [updated September 2009]. The Cochrane Collaboration, 2009. Available from www.cochrane-handbook.org.; 2009.
24. Centers for Disease Control and Prevention. Managing Acute Gastroenteritis Among Children: Oral Rehydration, Maintenance, and Nutritional Therapy. Pediatrics 2004;114:507.
25. Uhlig U, Pfeil N, Gelbrich G, et al. Dimenhydrinate in children with infectious gastroenteritis: a prospective, RCT. Pediatrics 2009;124:e622-32.
26. Pfeil N, Uhlig U, Kostev K, et al. Antiemetic medications in children with presumed infectious gastroenteritis--pharmacoepidemiology in Europe and Northern America. J Pediatr 2008;153:659-62, 62 e1-3.
27. Gouin S, Vo T, Roy M, Lebel D, Gravel J. A randomized double-blind trial comparing the effects of oral dimenhydrinate versus placebo in children with moderate vomiting from acute gastroenteritis [abstract]. In: Pediatric Academic Society's Annual Meeting. Denver, CO; 2011.
28. Moritz ML, Ayus JC. Improving intravenous fluid therapy in children with gastroenteritis. Pediatr Nephrol 2010;25:1383-4.
29. Moritz ML, Ayus JC. Prevention of hospital-acquired hyponatremia: a case for using isotonic saline. Pediatrics 2003;111:227-30.
30. Moritz ML, Ayus JC. Hypotonic fluids should not be used in volume-depleted children. Kidney Int 2005;68:409-10.
31. Gregorio L, Sutton CL, Lee DA. Central pontine myelinolysis in a previously healthy 4-year-old child with acute rotavirus gastroenteritis. Pediatrics 1997;99:738-43.
32. Moritz ML, Ayus JC. Preventing neurological complications from dysnatremias in children. Pediatr Nephrol 2005;20:1687-700.
33. Playfor S. Fatal iatrogenic hyponatraemia. Arch Dis Child 2003;88:646-7.
34. Hanna M, Saberi MS. Incidence of hyponatremia in children with gastroenteritis treated with hypotonic intravenous fluids. Pediatr Nephrol 2010;25:1471-5.
35. Neville KA, Verge CF, O'Meara MW, Walker JL. High antidiuretic hormone levels and hyponatremia in children with gastroenteritis. Pediatrics 2005;116:1401-7.
36. Beck CE. Hypotonic versus isotonic maintenance intravenous fluid therapy in hospitalized children: a systematic review. Clin Pediatr (Phila) 2007;46:764-70.
37. Freedman SB, Thull-Freedman JD. Vomiting, diarrhea, and dehydration in children. In: Tintinalli JE, Stapczynski JS, Ma OJ, Cline DM, Cydulka RK, eds. Tintinalli's Emergency Medicine: A Comprehensive Study Guide. 7th ed. New York ,NY: The McGraw-Hill Companies Inc.; 2011:830-8.
38. Chen L, Hsiao A, Langhan M, Riera A, Santucci KA. Use of bedside ultrasound to assess degree of dehydration in children with gastroenteritis. Acad Emerg Med 2010;17:1042-7.
39. Friedman JN, Goldman RD, Srivastava R, Parkin PC. Development of a clinical dehydration scale for use in children between 1 and 36 months of age. J Pediatr 2004;145:201-7.
40. Bailey B, Gravel J, Goldman RD, Friedman JN, Parkin PC. External validation of the clinical dehydration scale for children with acute gastroenteritis. Acad Emerg Med 2010;17:583-8.
41. Gravel J, Manzano S, Guimont C, Lacroix L, Gervaix A, Bailey B. [Multicenter validation of the clinical dehydration scale for children]. Arch Pediatr 2010;17:1645-51.
42. Goldman RD, Friedman JN, Parkin PC. Validation of the clinical dehydration scale for children with acute gastroenteritis. Pediatrics 2008;122:545-9.
43. Levine AC, Shah SP, Umulisa I, et al. Ultrasound assessment of severe dehydration in children with diarrhea and vomiting. Acad Emerg Med 2010;17:1035-41.
44. Kosiak W, Swieton D, Piskunowicz M. Sonographic inferior vena cava/aorta diameter index, a new approach to the body fluid status assessment in children and young adults in emergency ultrasound--preliminary study. Am J Emerg Med 2008;26:320-5.
45. Steiner MJ, DeWalt DA, Byerley JS. Is this child dehydrated? JAMA 2004;291:2746-54.

46. Levy JA, Bachur RG. Bedside ultrasound in the pediatric emergency department. Curr Opin Pediatr 2008;20:235-42.
47. Buscemi N, Hartling L, Vandermeer B, Tjosvold L, Klassen TP. Single data extraction generated more errors than double data extraction in systematic reviews. J Clin Epidemiol 2006;59:697-703.
48. Shea BJ, Hamel C, Wells GA, et al. AMSTAR is a reliable and valid measurement tool to assess the methodological quality of systematic reviews. J Clin Epidemiol 2009;62:1013-20.
49. Higgins JPT, Green S. (editors). Cochrane Handbook for Systematic Reviews of Interventions Version 5.0.2 [updated September 2009]. The Cochrane Collaboration. [cited 2010 Nov 29]. Available from www.cochrane-handbook.org.
50. Composition of macro geographical (continental) regions, geographical sub-regions, and selected economic and other groupings (footnote c). United Nations Statistics Division. revised February 17, 2011. (Accessed March 4, 2011, at http://unstats.un.org/unsd/methods/m49/m49regin.htm#ftnc.)
51. U.S. Department of Health & Human Services. Child Health Care Quality Toolbox: Established Child Health Care Quality Measures – AHRQ Quality Indicators. Agency for Healthcare Research and Quality, 2006. (Accessed November 29, 2010, at http://www.ahrq.gov/chtoolbx/measure3.htm.)
52. CIHR-President's Advisory Board, Strategy for Patient-Oriented Research. 2010. (Accessed March 7, 2011, at http://www.irsc.gc.ca/e/41232.html#d5.)
53. Deeks JJ, Altman DG, Bradburn MJ. Statistical methods for examining heterogeneity and combining results from several studies in meta-analysis. In: Egger M, Davey Smith G, Altman DG, eds. Systematic reviews in health care: meta-analysis in context. London: BMJ Publication Group; 2001.
54. Petitti DB. Approaches to heterogeneity in meta-analysis. Stat Med 2001;20:3625-33.
55. Bucher HC, Guyatt GH, Griffith LE, Walter SD. The results of direct and indirect treatment comparisons in meta-analysis of randomized controlled trials. J Clin Epidemiol 1997;50:683-91.
56. Higgins JP, Thompson SG. Quantifying heterogeneity in a meta-analysis. Stat Med 2002;21:1539-58.
57. Higgins JP, Thompson SG, Deeks JJ, Altman DG. Measuring inconsistency in meta-analyses. BMJ 2003;327:557-60.
58. Light RJ, Pillemer D. Summing up: the science of review research. Cambridge, MA: Harvard University Press; 1984.
59. Begg CB, Mazumdar M. Operating characteristics of a rank correlation test for publication bias. Biometrics 1994;50:1088-101.
60. Egger M, Davey Smith G, Schneider M, Minder C. Bias in meta-analysis detected by a simple, graphical test. BMJ 1997;315:629-34.
61. Duval S, Tweedie R. Trim and fill: A simple funnel-plot-based method of testing and adjusting for publication bias in meta-analysis. Biometrics 2000;56:455-63.
62. The GRADE Working Group. GRADE handbook for grading quality of evidence and strength of recommendation. Version 3.2 ed. 2009.
63. Whiting P, Rutjes AW, Reitsma JB, Bossuyt PM, Kleijnen J. The development of QUADAS: a tool for the quality assessment of studies of diagnostic accuracy included in systematic reviews. BMC Med Res Methodol 2003;3:25.
64. Reitsma JB, Rutjes AWS, Whiting P, Vlassov VV, Leeflang MMG, Deeks JJ. Assessing methodological quality. In: Deeks JJ, Bossuyt PM, Gatsonis C, eds. Cochrane Handbook for Systematic Reviews of Diagnostic Test Accuracy; Version 1.0.0. The Cochrane Collaboration. 2009. Available from: http://srdta.cochrane.org/.
65. Macaskill P, Gatsonis C, Deeks JJ, Harbord RM, Takwoingi Y. Analysing and Presenting Results. In: Deeks JJ, Bossuyt PM, Gatsonis C, eds. Cochrane Handbook for Systematic Reviews of Diagnostic Test Accuracy; Version 1.0. The Cochrane Collaboration. 2010. Available from: http://srdta.cochrane.org/.
66. The AGREE Collaboration. Appraisal of Guidelines for Research & Evaluation (AGREE) Instrument [website on the Internet]. (Accessed December 1, 2010, at www.agreecollaboration.org.)

67. Moher D, Cook DJ, Eastwood S, Olkin I, Rennie D, Stroup DF. Improving the quality of reports of meta-analyses of randomised controlled trials: the QUOROM statement. Quality of Reporting of Meta-analyses. Lancet 1999;354:1896-900.
68. NIH Consensus Development Program [website on the Internet]. (Accessed December 1, 2010, at http://consensus.nih.gov/ABOUTCDP.htm.)
69. Klassen TP, Wiebe N, Russell K, et al. Abstracts of randomized controlled trials presented at the society for pediatric research meeting: an example of publication bias. Arch Pediatr Adolesc Med 2002;156:474-9.
70. Moher D, Fortin P, Jadad AR, et al. Completeness of reporting of trials published in languages other than English: implications for conduct and reporting of systematic reviews. Lancet 1996;347:363-6.
71. Moher D, Cook DJ, Jadad AR, et al. Assessing the quality of reports of randomised trials: implications for the conduct of meta-analyses. Health Technol Assess 1999;3:i-iv, 1-98.
72. Moher D, Pham B, Jones A, et al. Does quality of reports of randomised trials affect estimates of intervention efficacy reported in meta-analyses? Lancet 1998;352:609-13.
73. Moher D, Pham B, Klassen TP, et al. What contributions do languages other than English make on the results of meta-analyses? J Clin Epidemiol 2000;53:964-72.
74. Sampson M, Barrowman NJ, Moher D, et al. Should meta-analysts search Embase in addition to Medline? J Clin Epidemiol 2003;56:943-55.
75. Pham B, Platt R, McAuley L, Klassen TP, Moher D. Is there a "best" way to detect and minimize publication bias? An empirical evaluation. Eval Health Prof 2001;24:109-25.
76. Wiebe N, Vandermeer B, Platt RW, Klassen TP, Moher D, Barrowman NJ. A systematic review identifies a lack of standardization in methods for handling missing variance data. J Clin Epidemiol 2006;59:342-53.
77. Crumley ET, Wiebe N, Cramer K, Klassen TP, Hartling L. Which resources should be used to identify RCT/CCTs for systematic reviews: a systematic review. BMC Med Res Methodol 2005;5:24.
78. Freedman SB, Powell EC, Nava-Ocampo AA, Finkelstein Y. Ondansetron dosing in pediatric gastroenteritis: a prospective cohort, dose-response study. Paediatr Drugs 2010;12:405-10.
79. Freedman SB, Eltorky M, Gorelick M. Evaluation of a gastroenteritis severity score for use in outpatient settings. Pediatrics 2010;125:e1278-85.
80. Freedman SB, Deiratany S, Goldman RD, Benseler S. Development of a Caregiver Gastroenteritis Knowledge Questionnaire. Ambul Pediatr 2008;8:261-5.
81. Freedman SB, Adler M, Seshadri R, Powell EC. Oral ondansetron for gastroenteritis in a pediatric emergency department. N Engl J Med 2006;354:1698-705.
82. Shavit I, Brant R, Nijssen-Jordan C, Galbraith R, Johnson DW. A novel imaging technique to measure capillary-refill time: improving diagnostic accuracy for dehydration in young children with gastroenteritis. Pediatrics 2006;118:2402-8.
83. Graham JM, Fitzpatrick EA, Black KJ. "My child can't keep anything down!" Interviewing parents who bring their preschoolers to the emergency department for diarrhea, vomiting, and dehydration. Pediatr Emerg Care 2010;26:251-6.
84. Freedman SB, Couto M, Spooner L, Haladyn JK. The implementation of a gastroenteritis education program. Am J Emerg Med 2011;29:271-7.
85. Freedman SB, Parkin PC, Willan AR, Schuh S. Rapid versus Standard Intravenous Rehydration in Paediatric Gastroenteritis: A Pragmatic, Blinded, Randomised Clinical Trial BMJ 2011;In press.
86. Bellemare S, Hartling L, Wiebe N, et al. Oral rehydration versus intravenous therapy for treating dehydration due to gastroenteritis in children: a meta-analysis of randomised controlled trials. BMC Med 2004;2:11.
87. Kaur S, Freedman SB. Acute Gastroenteritis: In Children Aged 2 Months Through 5 Years. The Hospital for Sick Children, Toronto. Accessed November 29, 2010. Available at: http://notesapp01/hsc/policies.nsf/c74cce2b9811db728525697b0072cdc5/179a6f4e8a7f6f318525764a004c2eb3?OpenDocument&Highlight=0,gastroenteritis.

88. Perlstein PH, Lichtenstein P, Cohen MB, et al. Implementing an evidence-based acute gastroenteritis guideline at a children's hospital. Jt Comm J Qual Improv 2002;28:20-30.
89. McLeod L, French B, Dai D, Localio R, Keren R. Patient volume and quality of care for young children hospitalized with acute gastroenteritis. Arch Pediatr Adolesc Med 2011;165:857-63.
90. Tieder JS, Robertson A, Garrison MM. Pediatric hospital adherence to the standard of care for acute gastroenteritis. Pediatrics 2009;124:e1081-7.
91. Jain S, Elon LK, Johnson BA, Frank G, Deguzman M. Physician practice variation in the pediatric emergency department and its impact on resource use and quality of care. Pediatr Emerg Care 2010;26:902-8.
92. Lougheed MD, Garvey N, Chapman KR, et al. Variations and gaps in management of acute asthma in Ontario emergency departments. Chest 2009;135:724-36.
93. Plint AC, Johnson DW, Wiebe N, et al. Practice variation among pediatric emergency departments in the treatment of bronchiolitis. Acad Emerg Med 2004;11:353-60.
94. Goldman RD, Scolnik D, Chauvin-Kimoff L, et al. Practice variations in the treatment of febrile infants among pediatric emergency physicians. Pediatrics 2009;124:439-45.
95. Plint A, Clifford T, Perry J, et al. Wrist buckle fractures: a survey of current practice patterns and attitudes toward immobilization. CJEM 2003;5:95-100.
96. Richer LP, Laycock K, Millar K, et al. Treatment of children with migraine in emergency departments: national practice variation study. Pediatrics 2010;126:e150-5.
