# Supplementary material for: Gastroenteritis Therapies in Developed Countries: Systematic Review and Meta-Analysis
Source: PLoS One. 2015 Jun 15;10(6):e0128754. doi: 10.1371/journal.pone.0128754 (PMC4468143; doi:10.1371/journal.pone.0128754)
Supplement: S2 File — (DOC) [file pone.0128754.s002.doc]

**S2 File Search Strategy**

Database: Medline via Ovid <1946 to Present>

Search Title: Gastroenteritis KT Grant 1.2 – RCTs

*Terms for Gastroenteritis:*

1. exp Gastroenteritis/

2. Rotavirus/

3. Rotavirus Infections/

4. exp Norovirus/

5. exp Diarrhea/

6. Vomiting/

7. Dehydration/

8. gastroenterit*.tw.

9. (diarrhea or diarrhoea).tw.

10. vomit*.tw.

11. dehydrat*.tw.

12. gastritis.tw.

13. **or/1-12** [combination of MeSH and text words for Gastro] (290,904)

*Terms for Oral, IV, and Nasogastric Rehydration:*

14. exp fluid therapy/

15. Rehydration Solutions/

16. Administration, Oral/

17. Infusions, Intravenous/

18. Enteral Nutrition/

19. Infusions, Parenteral/

20. exp Parenteral Nutrition/

21. Parenteral Nutrition Solutions/

22. rehydrat*.tw.

23. (ORT or ORS).tw.

24. ((fluid or IV) adj3 therap*).tw.

25. (water adj2 electrolyte adj2 balance).tw.

26. ((fluid* or IV or intravenous or enteral or parenteral) adj3 therap*).tw.

27. ((fluid* or IV or intravenous or enteral or parenteral) adj3 infusion*).tw.

28. ((fluid* or IV or intravenous or enteral or parenteral) adj3 drip*).tw.

29. (nasogastric* or (NG adj3 tube*)).tw.

30. **or/14-29** [combination of MeSH and text words for rehydration, oral or other] (267,422)

*Terms for Treatment with Antiemetics:*

31. exp Antiemetics/

32. exp Dopamine Antagonists/

33. exp Serotonin Antagonists/

34. exp Cholinergic Antagonists/

35. exp Histamine Antagonists/

36. exp Benzodiazepines/

37. exp Adrenal Cortex Hormones/

38. exp Cannabinoids/

39. (antiemetic* or anti-emetic*).tw.

40. anti-vomit*.tw.

41. ((dopamine or serotonin or cholinergic or histamine) adj1 antagonist*).tw.

42. benzodiazepine*.tw.

43. "adrenal cortex hormone*".tw.

44. cannabinoid*.tw.

45. ("5-HT3" or "5-hydroxytryptamine").tw.

46. **or/31-45** [MeSH and textwords for antiemetics] (607,915)

*Terms for Treatment with Probiotics:*

47. Probiotics/

48. exp Lactobacillaceae/

49. exp Lactococcus/

50. exp Enterococcaceae/

51. exp Streptococcaceae/

52. exp Saccharomyces/

53. Probiotic*.tw.

54. Lactobacill*.tw.

55. Lactococc*.tw.

56. Enterococc*.tw.

57. Streptococc*.tw.

58. Saccharomyc*.tw.

59. bifidobacter*.tw.

60. (microbi* adj3 supplement*).tw.

61. ("L. GG" or "L.GG").tw.

62. **or/47-61** [MeSH and textwords for probiotics] (222,663)

*Terms for Child (≤18 years) Population:*

63. exp Child/

64. exp Infant/

65. exp pediatrics/

66. Adolescent/

67. Minors/

68. (infant* or child* or adolescen* or teen* or youth* or young or p?ediatric* or juvenile*).tw. (1,425,360)

69. **or/63-68** [combination of MeSH and text terms for children] (3,062,183)

*Filter for RCTs:*

70. randomized controlled trial.pt.

71. controlled clinical trial.pt.

72. randomized.ab.

73. placebo.ab.

74. clinical trials as topic.sh.

75. randomly.ab.

76. trial.ti.

77. or/70-76 (774,732)

78. exp animals/ not humans.sh.

79. **77 not 78** [Cochrane RCT search strategy to max sensitivity and precision] (713,951)

*Results for Rehydration Therapy:*

80. **and/13,30,69,79** [gastro + rehydration + children + RCTs] (1,756)

81. limit 80 to humans (1,750)

82. remove duplicates from 81 (1,722)

*Results for Treatment with Anti-emetic:*

83. **and/13,46,69,79** [gastro + antiemetics + children + RCTs] (1,403)

84. limit 83 to humans (1,402)

85. limit 84 to yr="2006 -Current" (296)

86. remove duplicates from 85 (283)

*Results for Treatment with Probiotics:*

87. and/13,62,69,79 [gastro + probiotics + children + RCTs] (462)

88. limit 87 to humans (460)

89. limit 88 to yr="2006 -Current" (211)

90. remove duplicates from 89 (193)

Database: Medline via Ovid <1946 to Present>

Search Title: Gastroenteritis KT Grant 1.4--IV Flow terms ANDed in | Medline

*Terms for Gastroenteritis:*

1. exp Gastroenteritis/

2. Rotavirus/

3. Rotavirus Infections/

4. exp Norovirus/

5. exp Diarrhea/

6. Vomiting/

7. Dehydration/

8. gastroenterit*.tw.

9. (diarrhea or diarrhoea).tw.

10. vomit*.tw.

11. dehydrat*.tw.

12. gastritis.tw.

13. **or/1-12** [combination of MeSH and text words for Gastro] (291,101)

*Terms for IV therapy:*

14. Infusions, Intravenous/

15. Enteral Nutrition/

16. Infusions, Parenteral/

17. exp Parenteral Nutrition/

18. ((fluid or IV) adj3 therap*).tw.

19. ((fluid* or IV or intravenous or enteral or parenteral) adj3 therap*).tw.

20. ((fluid* or IV or intravenous or enteral or parenteral) adj3 infusion*).tw.

21. ((fluid* or IV or intravenous or enteral or parenteral) adj3 drip*).tw.

22. **or/14-21** [combination of terms for IVT] (139,452)

*Terms for Rates and Compositions of Fluids:*

23. exp fluid therapy/

24. Drug administration schedule/

25. exp Hypertonic Solutions/

26. Hypotonic Solutions/

27. Isotonic Solutions/

28. Rehydration Solutions/

29. Pharmaceutical Solutions/

30. Osmolar Concentration/

31. Electrolytes/

32. rehydrat*.tw.

33. ((saline or dextrose or ringer's) adj3 (solution* or infusions* or bolus)).tw.

34. (rate* adj3 (flow* or infusion* or dr?p*)).tw.

35. (fluid* adj3 (balance* or composition* or concentration*)).tw.

36. **or/23-35** [MeSH and keywords for fluid rates/composition] (261,975)

*Terms for Child (≤18 years) Population:*

37. exp child/

38. exp infant/

39. exp pediatrics/

40. adolescent/

41. minors/

42. (infant* or child* or adolescen* or teen* or youth* or young or p?ediatric* or juvenile*).tw.

43. **or/37-42** [MeSH and keywords for children] (3,064,055)

*Filter for RCTs:*

44. randomized controlled trial.pt.

45. controlled clinical trial.pt.

46. randomized.ab.

47. placebo.ab.

48. clinical trials as topic.sh.

49. randomly.ab.

50. trial.ti.

51. or/44-50

52. exp animals/ not humans.sh.

53. **51 not 52** [Cochrane RCT filter to maximize sensitivity/precision] (713,951)

54. **and/13,22,36,43,53** [gastro + IVT + rates/composition + child + RCTs] (171)

55. limit 54 to humans (170)

56. remove duplicates from 55 (165)
