# Supplementary material for: Gastroenteritis Therapies in Developed Countries: Systematic Review and Meta-Analysis
Source: PLoS One. 2015 Jun 15;10(6):e0128754. doi: 10.1371/journal.pone.0128754 (PMC4468143; doi:10.1371/journal.pone.0128754)
Supplement: S1 Table — (DOC) [file pone.0128754.s003.doc]

**S1 Table Risk of Bias**

| **Author Year** | **Random sequence generation** | **Allocation concealment** | **Blinding of participants and personnel** | **Blinding of outcome assessment** | **Incomplete outcome data** | **Selective outcome reporting** | **Other sources of bias** | **Overall risk of bias** |
| --- | --- | --- | --- | --- | --- | --- | --- | --- |
| **Intravenous Therapy vs. Oral Rehydration Therapy** | | | | | | | | |
| Atherly-John 2002[^1^](#_ENREF_1) | unclear | low | unclear | low | low | low | low | unclear |
| Gremse 1995[^2^](#_ENREF_2) | unclear | unclear | unclear | unclear | low | low | unclear | unclear |
| Issenman 1993[^3^](#_ENREF_3) | unclear | unclear | unclear | unclear | low | low | unclear | unclear |
| Listernick 1986[^4^](#_ENREF_4) | unclear | unclear | unclear | unclear | low | low | unclear | unclear |
| Mackenzie 1991[^5^](#_ENREF_5) | unclear | unclear | unclear | unclear | low | low | low | unclear |
| Nager 2002[^6^](#_ENREF_6) | unclear | unclear | unclear | unclear | low | low | unclear | unclear |
| Santosham 1982[^7^](#_ENREF_7) | unclear | unclear | unclear | unclear | low | low | unclear | unclear |
| Spandorfer 2005[^8^](#_ENREF_8) | low | low | low | low | low | low | low | low |
| Tamer 1985[^9^](#_ENREF_9) | unclear | unclear | unclear | unclear | low | low | unclear | unclear |
| Vesikari 1987[^10^](#_ENREF_10) | low | unclear | unclear | unclear | low | low | low | unclear |
| **Antiemetics** | | | | | | | | |
| Freedman 2006[^11^](#_ENREF_11) | low | low | low | low | low | low | low | low |
| Gouin 2011[^12^](#_ENREF_12) | low | low | low | low | low | low | low | low |
| Norton 2011[^13^](#_ENREF_13) | unclear | unclear | unclear | unclear | unclear | unclear | unclear | unclear |
| Qazi 2014[^14^](#_ENREF_14) | low | low | low | low | unclear | low | low | unclear |
| Ramsook 2002[^15^](#_ENREF_15) | low | low | low | low | low | low | unclear | unclear |
| Reeves 2002[^16^](#_ENREF_16) | low | low | low | low | low | low | unclear | unclear |
| Roslund 2008[^17^](#_ENREF_17) | unclear | unclear | low | low | low | low | low | unclear |
| Stork 2006[^18^](#_ENREF_18) | low | low | low | low | low | low | unclear | unclear |
| Uhlig 2009[^19^](#_ENREF_19) | unclear | unclear | unclear | unclear | low | low | low | unclear |
| **Probiotics** | | | | | | | | |
| Canani 2007[^20^](#_ENREF_20) | low | unclear | unclear | low | low | low | low | unclear |
| Henker 2007[^21^](#_ENREF_21) | low | unclear | unclear | unclear | low | low | unclear | unclear |
| Henker 2008[^22^](#_ENREF_22) | low | unclear | unclear | unclear | low | low | unclear | unclear |
| Nixon 2012[^23^](#_ENREF_23) | low | low | low | low | unclear | low | low | unclear |
| Passariello 2012[^24^](#_ENREF_24) | low | low | low | low | low | low | low | low |
| Ritchie 2010[^25^](#_ENREF_25) | low | low | low | low | low | low | low | low |
| **Intravenous Fluid Rates & Compositions** | | | | | | | | |
| Allen 2014[^26^](#_ENREF_26) | unclear | unclear | unclear | unclear | low | low | high | high |
| Freedman 2011[^27^](#_ENREF_27) |  | low | low | low | low | low | low | low |
| Levy 2013[^28^](#_ENREF_28) | low | low | low | low | low | low | low | low |
| Nager 2010[^29^](#_ENREF_29) | low | unclear | unclear | unclear | low | low | low | unclear |
| Neville 2006[^30^](#_ENREF_30) | unclear | unclear | unclear | unclear | unclear | low | low | unclear |
| Powell 2011[^31^](#_ENREF_31) | low | low | unclear | unclear | low | low | low | unclear |

1. Atherly-John YC, Cunningham SJ, Crain EF. A randomized trial of oral vs intravenous rehydration in a pediatric emergency department. Arch Pediatr Adolesc Med 2002;156:1240-3.

2. Gremse DA. Effectiveness of nasogastric rehydration in hospitalized children with acute diarrhea. J Pediatr Gastroenterol Nutr 1995;21:145-8.

3. Issenman RM, Leung AK. Oral and intravenous rehydration of children. Can Fam Physician 1993;39:2129-36.

4. Listernick R, Zieserl E, Davis AT. Outpatient oral rehydration in the United States. Am J Dis Child 1986;140:211-5.

5. Mackenzie A, Barnes G. Randomised controlled trial comparing oral and intravenous rehydration therapy in children with diarrhoea. BMJ 1991;303:393-6.

6. Nager AL, Wang VJ. Comparison of nasogastric and intravenous methods of rehydration in pediatric patients with acute dehydration. Pediatrics 2002;109:566-72.

7. Santosham M, Daum RS, Dillman L, et al. Oral rehydration therapy of infantile diarrhea: a controlled study of well-nourished children hospitalized in the United States and Panama. N Engl J Med 1982;306:1070-6.

8. Spandorfer PR, Alessandrini EA, Joffe MD, Localio R, Shaw KN. Oral versus intravenous rehydration of moderately dehydrated children: a randomized, controlled trial. Pediatrics 2005;115:295-301.

9. Tamer AM, Friedman LB, Maxwell SR, Cynamon HA, Perez HN, Cleveland WW. Oral rehydration of infants in a large urban U.S. medical center. J Pediatr 1985;107:14-9.

10. Vesikari T, Isolauri E, Baer M. A comparative trial of rapid oral and intravenous rehydration in acute diarrhoea. Acta Paediatr Scand 1987;76:300-5.

11. Freedman SB, Adler M, Seshadri R, Powell EC. Oral ondansetron for gastroenteritis in a pediatric emergency department. N Engl J Med 2006;354:1698-705.

12. Gouin S, Vo T, Roy M, Lebel D, Gravel J. A randomized double-blind trial comparing the effects of oral dimenhydrinate versus placebo in children with moderate vomiting from acute gastroenteritis [abstract]. Pediatric Academic Society's Annual Meeting. Denver, CO2011.

13. Norton I. Ondansetron wafers for paediatric nausea and vomiting in an Australlan ED; a randomised controlled trial. Australasian College for Emergency Medicine 18th Annual Scientific Meeting. Sydney, Australia: Emergency Medicine Australasia; 2011:19.

14. Qazi K, BinSalleeh HM, Shah UH, et al. Effectiveness of granisetron in controlling pediatric gastroenteritis-related vomiting after discharge from the ED. Am J Emerg Med 2014;32:1046-50.

15. Ramsook C, Sahagun-Carreon I, Kozinetz CA, Moro-Sutherland D. A randomized clinical trial comparing oral ondansetron with placebo in children with vomiting from acute gastroenteritis. Ann Emerg Med 2002;39:397-403.

16. Reeves JJ, Shannon MW, Fleisher GR. Ondansetron decreases vomiting associated with acute gastroenteritis: a randomized, controlled trial. Pediatrics 2002;109:e62.

17. Roslund G, Hepps TS, McQuillen KK. The role of oral ondansetron in children with vomiting as a result of acute gastritis/gastroenteritis who have failed oral rehydration therapy: a randomized controlled trial. Ann Emerg Med 2008;52:22-9 e6.

18. Stork CM, Brown KM, Reilly TH, Secreti L, Brown LH. Emergency department treatment of viral gastritis using intravenous ondansetron or dexamethasone in children. Acad Emerg Med 2006;13:1027-33.

19. Uhlig U, Pfeil N, Gelbrich G, et al. Dimenhydrinate in children with infectious gastroenteritis: a prospective, RCT. Pediatrics 2009;124:e622-32.

20. Canani RB, Cirillo P, Terrin G, et al. Probiotics for treatment of acute diarrhoea in children: randomised clinical trial of five different preparations. BMJ 2007;335:340.

21. Henker J, Laass M, Blokhin BM, et al. The probiotic Escherichia coli strain Nissle 1917 (EcN) stops acute diarrhoea in infants and toddlers. Eur J Pediatr 2007;166:311-8.

22. Henker J, Laass MW, Blokhin BM, et al. Probiotic Escherichia coli Nissle 1917 versus placebo for treating diarrhea of greater than 4 days duration in infants and toddlers. Pediatr Infect Dis J 2008;27:494-9.

23. Nixon AF, Cunningham SJ, Cohen HW, Crain EF. The effect of Lactobacillus GG on acute diarrheal illness in the pediatric emergency department. Pediatr Emerg Care 2012;28:1048-51.

24. Passariello A, Terrin G, Cecere G, et al. Randomised clinical trial: efficacy of a new synbiotic formulation containing Lactobacillus paracasei B21060 plus arabinogalactan and xilooligosaccharides in children with acute diarrhoea. Aliment Pharmacol Ther 2012;35:782-8.

25. Ritchie BK, Brewster DR, Tran CD, Davidson GP, McNeil Y, Butler RN. Efficacy of Lactobacillus GG in aboriginal children with acute diarrhoeal disease: a randomised clinical trial. J Pediatr Gastroenterol Nutr 2010;50:619-24.

26. Allen CH, Goldman RD, Simon HK, et al. Balanced Crystalloid or Saline in Pediatric Gastroenteritis: A Randomized Controlled Trial. SAEM Annual Meeting Abstracts Academic Emergency Medicine, 21: S5–S327 doi: 101111/acem123652014.

27. Freedman SB, Parkin PC, Willan AR, Schuh S. Rapid versus standard intravenous rehydration in paediatric gastroenteritis: pragmatic blinded randomised clinical trial. BMJ 2011;343:d6976.

28. Levy JA, Bachur RG, Monuteaux MC, Waltzman M. Intravenous dextrose for children with gastroenteritis and dehydration: a double-blind randomized controlled trial. Ann Emerg Med 2013;61:281-8.

29. Nager AL, Wang VJ. Comparison of ultrarapid and rapid intravenous hydration in pediatric patients with dehydration. Am J Emerg Med 2010;28:123-9.

30. Neville KA, Verge CF, Rosenberg AR, O'Meara MW, Walker JL. Isotonic is better than hypotonic saline for intravenous rehydration of children with gastroenteritis: a prospective randomised study. Arch Dis Child 2006;91:226-32.

31. Powell CV, Priestley SJ, Young S, Heine RG. Randomized clinical trial of rapid versus 24-hour rehydration for children with acute gastroenteritis. Pediatrics 2011;128:e771-8.
