# Supplementary material for: Gastroenteritis Therapies in Developed Countries: Systematic Review and Meta-Analysis
Source: PLoS One. 2015 Jun 15;10(6):e0128754. doi: 10.1371/journal.pone.0128754 (PMC4468143; doi:10.1371/journal.pone.0128754)
Supplement: S2 Table — (DOC) [file pone.0128754.s004.doc]

**S2 Table Baseline Characteristics - Intravenous vs Oral Rehydration Therapy**

| **Study** | **Country of Study; Years** | **Enrollment Criteria‡** | **Number of patients** | **Age, Mos: Mean (SD) or Median [Range]** | **Duration of Symptoms, Hours: Mean (SD) or Median [range]** | **Method of Dehydration Assessment** | **Dehydration Severity†** | | | **Vomiting*** |
| --- | --- | --- | --- | --- | --- | --- | --- | --- | --- | --- |
|  |  |  |  |  |  |  | **%**  **Mild** | **% Moderate** | **% Severe** |  |
| Atherly-John[^1^](#_ENREF_1) | USA; NR | - 3 months – 17 years - Previously healthy - <1 week of AGE - Moderate dehydration | 34 | 59.4 (NR) | - | Required to have ≥4 standard published criteria for moderate dehydration[^2^](#_ENREF_2) | - | - | - | - |
| Gremse[^3^](#_ENREF_3) | USA; 1991-93 | - 2 months – 2 years - Previously healthy - >5^th^ percentile weight for age - <5 days AGE - ORT was unsuccessful - Hospitalized for moderate (5-10%) dehydration | 24 | 8.9 (1.6) | 56.4 (10.8) | 5, 7, 10% dehydration determined by clinical criteria[^4^](#_ENREF_4) | 6 | 0 | 0 | - |
| Issenman[^5^](#_ENREF_5) | Canada; NR | - 6 months – 31 months - Previously healthy - Mild to moderate (5-10%) dehydration - >5^th^ percentile weight for age - <5 days AGE | 42 | 15.1 (1.5) | 66 (36) | Determined by clinical criteria[^6^](#_ENREF_6) | - | - | - | 96% |
| Listernick[^7^](#_ENREF_7) | USA; 1983-84 | - 3 months – 24 months - Previously healthy - >5^th^ percentile weight for age - <5 days AGE - No antibiotics within 7 days - Dehydrated | 29 | 12.3 (5.8) | 54 (27.6) | Assessed based on vital signs, capillary refill, tears, mucous membranes, urinary output, general behaviour, previous weights | - | - | - | 5 (2.25) |
| Mackenzie[^8^](#_ENREF_8) | Australia; 1986-88 | - 3 months – 36 months - Previously healthy - <1 week of AGE - Moderate dehydration | 111 | 20 [12-26] | 66 [48-96] | Royal Children’s Hospital guidelines | 34 | 19 | 0 | 91% |
| Nager[^11^](#_ENREF_11) | USA; 1997-99 | - 3 months – 36 months - Previously healthy - <1 week of AGE - Moderate dehydration - Unable to tolerate oral fluids | 96 | - | - | Determined by one of the study investigators using clinical parameters[^12^](#_ENREF_12) | - | - | - | - |
| Santosham[^13^](#_ENREF_13) | USA; 1977-81 | - 3 months – 24 months - >3^rd^ percentile weight for age - <5 days AGE - ≥5% dehydration | 52 | - | - | As determined by house officers | - | - | - | - |
| Spandorfer[^14^](#_ENREF_14) | USA; 2001-03 | - 8 weeks – 3 years - Previously healthy - Well nourished - <5 days AGE - Moderate (5-10%) dehydration | 73 | 15.75 [2.2-33.4] | - | 10-point dehydration scale[^15^](#_ENREF_15) | 4 | 0 | 0 | 8 [0-28] |
| Tamer[^16^](#_ENREF_16) | USA; 1981-83 | - 3 months – 33 months - Previously healthy - <10% dehydration | 100 | 8.35 (0.2) | 122.4 (60) | Estimated clinically | 11 | 21 | 11 | 89% |
| Vesikari[^17^](#_ENREF_17) | Finland; 1985 | - < 5 years - Dehydration | 38 | 21.05 (15.3) | 64.4 (33.6) | As determined by physician on duty based on acute weight loss calculated as difference between expected weight, and actual weight | 6 | 0 | 0 | - |

Mos, Months; NR, Not Reported; TX, Treatment; PLC, Placebo; AGE, Acute Gastroenteritis; ORT, Oral Rehydration Therapy.

* Percentage represents the percentage of patients who had vomited prior to enrolment; otherwise the number of vomiting episodes is described as a mean (standard deviation) or median [range] during the interval prior to enrolment.

†Dehydration Severity represents the severity assessment classification assigned by the study authors. Numbers do not add up to 100% as the percent without any evidence of dehydration is not listed.

‡Taken verbatim from manuscript.

1. Atherly-John YC, Cunningham SJ, Crain EF. A randomized trial of oral vs intravenous rehydration in a pediatric emergency department. Arch Pediatr Adolesc Med 2002;156:1240-3.

2. Adelman RD, Solhaug MJ. Pathophysiology of body fluids and fluid therapy. In: Nelson WE, Behrman RE, Kliegman RM, Arvin AM, eds. Textbook of Pediatrics. 15 ed. Philadelphia, PA: WB Saunders Co; 1996:206-10.

3. Gremse DA. Effectiveness of nasogastric rehydration in hospitalized children with acute diarrhea. J Pediatr Gastroenterol Nutr 1995;21:145-8.

4. Klish WJ. Use of oral fluids in treatment of diarrhea. Pediatr Rev 1985;7:27-30.

5. Issenman RM, Leung AK. Oral and intravenous rehydration of children. Can Fam Physician 1993;39:2129-36.

6. World Health Organization. Treatment and prevention of diarrheal diseases. Guide for use of primary health care personnel. Geneva: World Health Organization; Scientific Publication No. 336; 1977.

7. Listernick R, Zieserl E, Davis AT. Outpatient oral rehydration in the United States. Am J Dis Child 1986;140:211-5.

8. Mackenzie A, Barnes G. Randomised controlled trial comparing oral and intravenous rehydration therapy in children with diarrhoea. BMJ 1991;303:393-6.

9. Royal Children's Hospital. Residents handbook. Melbourne; Royal Children's Hospital, 1983:3.2.

10. Practical paediatrics. Melbourne: Churchill Livingstone; 482-90; 1986.

11. Nager AL, Wang VJ. Comparison of nasogastric and intravenous methods of rehydration in pediatric patients with acute dehydration. Pediatrics 2002;109:566-72.

12. Practice parameter: the management of acute gastroenteritis in young children. American Academy of Pediatrics, Provisional Committee on Quality Improvement, Subcommittee on Acute Gastroenteritis. Pediatrics 1996;97:424-35.

13. Santosham M, Daum RS, Dillman L, et al. Oral rehydration therapy of infantile diarrhea: a controlled study of well-nourished children hospitalized in the United States and Panama. N Engl J Med 1982;306:1070-6.

14. Spandorfer PR, Alessandrini EA, Joffe MD, Localio R, Shaw KN. Oral versus intravenous rehydration of moderately dehydrated children: a randomized, controlled trial. Pediatrics 2005;115:295-301.

15. Gorelick MH, Shaw KN, Murphy KO. Validity and reliability of clinical signs in the diagnosis of dehydration in children. Pediatrics 1997;99:E6.

16. Tamer AM, Friedman LB, Maxwell SR, Cynamon HA, Perez HN, Cleveland WW. Oral rehydration of infants in a large urban U.S. medical center. J Pediatr 1985;107:14-9.

17. Vesikari T, Isolauri E, Baer M. A comparative trial of rapid oral and intravenous rehydration in acute diarrhoea. Acta Paediatr Scand 1987;76:300-5.
