# Supplementary material for: Gastroenteritis Therapies in Developed Countries: Systematic Review and Meta-Analysis
Source: PLoS One. 2015 Jun 15;10(6):e0128754. doi: 10.1371/journal.pone.0128754 (PMC4468143; doi:10.1371/journal.pone.0128754)
Supplement: S3 Table — (DOC) [file pone.0128754.s005.doc]

**S3 Table. Baseline Characteristics – Antiemetics**

| **Study** | **Comparison** | **Country of Study; Years** | **Enrollment Criteria** | **Number of patients** | **Age, Mos: Mean (SD) or Median [range]** | **Duration of Symptoms, Hours: Mean (SD) Median [range]** | **Method of Dehydration Assessment** |
| --- | --- | --- | --- | --- | --- | --- | --- |
|
| Freedman[^1^](#_ENREF_1) | Ondansetron vs. Placebo | USA;  2005-05 | - 6 months – 10 years - ≥1 vomit within 4 hours of triage - ≥1 diarrhea during illness - Mild – moderate dehydration | 215 | 24.5 (20.5) | - | 7-item scale |
| Gouin[^2^](#_ENREF_2) | Dimenhydrinate vs. Placebo | Canada; 2005-10 | - 1 year – 12 years - >5 vomit within 12 hours - Some dehydration | 152 | 40.8 (32.4) | 25.1 (36.5) | Treating physician |
| Norton^*^[^3^](#_ENREF_3) | Ondansetron vs. Placebo | Australia; NR | - 3 months – 12 years | 392 | - | - | NR |
| Qazi[^4^](#_ENREF_4) | Granisetron vs. Placebo | Saudi Arabia; 2009-10 | - 6 months - 8 years - AGE less than 7 days - Mild to moderate dehydration | 165 | 29.4 (21.8) | 52.0 (39.2) | Treating physician |
| Ramsook[^5^](#_ENREF_5) | Ondansetron vs. Placebo | USA;  NR | - 6 months – 12 years - >5 vomit within 24 hours | 145 | - | - | NR |
| Reeves[^6^](#_ENREF_6) | Ondansetron vs. Placebo | USA;  1999-2000 | - 1 months – 22 years - >3 vomit within 24 hours - Determined to require IV rehydration - <7 days AGE | 107 | 57.9 (54.6) | - | NR |
| Roslund[^7^](#_ENREF_7) | Ondansetron vs. Placebo | USA;  2004-05 | - 1 year – 10 years - Mild – moderate dehydration - Failed ORT | 106 | 45.6 [12-127] | 36 [24-144] | Assessed by physician employing clinical features[^8^](#_ENREF_8) |
| Stork[^9^](#_ENREF_9) | Ondansetron vs. dexamethasone | USA;  1999-2005 | - 6 months – 12 years - >3 vomit within 24 hours - Mild – moderate dehydration - Failed ORT | 166 | 32.7 (32.4) | - | Assessed by physician employing clinical features[^10^](#_ENREF_10) |
| Uhlig[^11^](#_ENREF_11) | Dimenhydrinate vs. Placebo | Germany;  2005-07 | - 6 months – 6 years - <24 hours vomiting - ≥2 vomit within 12 hours - None – mild dehydration | 243 | 23.4 (18) | - | Body weight change and standardized assessment scale[^12^](#_ENREF_12) |

Mos, Months; NR, Not Reported; TX, Treatment; PLC, Placebo; vs, versus; Mod, Moderate; Sev, Severe; IV, Intravenous; AGE, Acute Gastroenteritis; ORT, Oral Rehydration Therapy.

**^*^** Abstract information only – no baseline data published

†Dehydration Severity represents the severity assessment classification assigned by the study authors. Numbers do not add up to 100% as the percent without any evidence of dehydration is not listed.

‡Numbers reported reflected the frequency of episodes prior to emergency department visit as reported by the authors.

- data not reported

1. Freedman SB, Adler M, Seshadri R, Powell EC. Oral ondansetron for gastroenteritis in a pediatric emergency department. N Engl J Med 2006;354:1698-705.

2. Gouin S, Vo T, Roy M, Lebel D, Gravel J. A randomized double-blind trial comparing the effects of oral dimenhydrinate versus placebo in children with moderate vomiting from acute gastroenteritis [abstract]. Pediatric Academic Society's Annual Meeting. Denver, CO2011.

3. Norton I. Ondansetron wafers for paediatric nausea and vomiting in an Australlan ED; a randomised controlled trial. Australasian College for Emergency Medicine 18th Annual Scientific Meeting. Sydney, Australia: Emergency Medicine Australasia; 2011:19.

4. Qazi K, BinSalleeh HM, Shah UH, et al. Effectiveness of granisetron in controlling pediatric gastroenteritis-related vomiting after discharge from the ED. Am J Emerg Med 2014;32:1046-50.

5. Ramsook C, Sahagun-Carreon I, Kozinetz CA, Moro-Sutherland D. A randomized clinical trial comparing oral ondansetron with placebo in children with vomiting from acute gastroenteritis. Ann Emerg Med 2002;39:397-403.

6. Reeves JJ, Shannon MW, Fleisher GR. Ondansetron decreases vomiting associated with acute gastroenteritis: a randomized, controlled trial. Pediatrics 2002;109:e62.

7. Roslund G, Hepps TS, McQuillen KK. The role of oral ondansetron in children with vomiting as a result of acute gastritis/gastroenteritis who have failed oral rehydration therapy: a randomized controlled trial. Ann Emerg Med 2008;52:22-9 e6.

8. Practice parameter: the management of acute gastroenteritis in young children. American Academy of Pediatrics, Provisional Committee on Quality Improvement, Subcommittee on Acute Gastroenteritis. Pediatrics 1996;97:424-35.

9. Stork CM, Brown KM, Reilly TH, Secreti L, Brown LH. Emergency department treatment of viral gastritis using intravenous ondansetron or dexamethasone in children. Acad Emerg Med 2006;13:1027-33.

10. Duggan C, Santosham M, Glass RI. The management of acute diarrhea in children: oral rehydration, maintenance, and nutritional therapy. Centers for Disease Control and Prevention. MMWR Recomm Rep 1992;41:1-20.

11. Uhlig U, Pfeil N, Gelbrich G, et al. Dimenhydrinate in children with infectious gastroenteritis: a prospective, RCT. Pediatrics 2009;124:e622-32.

12. World Health Organization. The treatment of diarrhea: a manual for physicians and other senior health workers. 4th revision. Geneva, Switzerland: World Health Organization; 2005.
