# Supplementary material for: Gastroenteritis Therapies in Developed Countries: Systematic Review and Meta-Analysis
Source: PLoS One. 2015 Jun 15;10(6):e0128754. doi: 10.1371/journal.pone.0128754 (PMC4468143; doi:10.1371/journal.pone.0128754)
Supplement: S4 Table — (DOC) [file pone.0128754.s006.doc]

**S4 Table. Baseline Characteristics – Probiotics**

| **Study** | **Comparison** | **Country of Study; Years** | **Enrollment Criteria** | **Number of patients** | **Age, Mos: Mean (SD) or Median [range]** | **Duration of Symptoms, Hours: Mean (SD) or Median [range]** | **Method of Dehydration Assessment** | **Dehydration Severity†** | | |
| --- | --- | --- | --- | --- | --- | --- | --- | --- | --- | --- |
|  |  |  |  |  |  |  |  | **%**  **Mild** | **%**  **Mild** | **%**  **Mild** |
| Canani [^1^](#_ENREF_1) | Multiple^1^ vs. Placebo | Italy;  1999-2000 | - 3 months – 36 months - ≥3 loose or liquid stools in a day - Diarrhea duration < 48 hours - Less than severe dehydration | 571 | 17.2 [9.5-27] | 9.8 [4-18] | NR | - | - | - |
| Henker [^2^](#_ENREF_2) | *E. coli* vs. Placebo | Ukraine;  2005 | - 0 – 4 years - ≥3 loose or liquid stools in a day - Diarrhea duration < 3 days - <5% dehydration | 113 | 22 (NR) | 36 (NR) | NR | - | - | - |
| Henker [^3^](#_ENREF_3) | *E. coli* vs. Placebo | Ukraine;  NR | - 3 months – 10 years - Acute watery diarrhea - Diarrhea duration < 7 days - Less than severe dehydration | 151 | 24.9 [1-47] | 5.8 (2.1) | NR | - | - | - |
| Nixon[^4^](#_ENREF_4) | *L. rhamnosus* vs. Placebo | USA;  2008-09 | - 6 months – 6 years - ≥3 loose or liquid stools in a day - Diarrhea duration ≤ 7 days | 155 | 21.3 (15.5) | 63.6 (30.0) | NR | - | - | - |
| Passariello [^5^](#_ENREF_5) | *L. paracasei* vs. Placebo | Italy;  2010-11 | - 3 months – 36 months - ≥3 loose or liquid stools in a day - Diarrhea duration ≤ 24 hours - Mild – moderate dehydration | 110 | 20.5 [16.7-24.3] | 8.4 [8-8.7] | Pediatricians estimated degree of dehydration using a seven-point Likert scale | 24 | 30 | 0 |
| Ritchie [^8^](#_ENREF_8) | *L. casei* vs. Placebo | Australia; 2002-04 | - 4 months – 24 months - ≥3 loose or liquid stools in a day - Diarrhea duration < 27 days - Tolerating ORT | 70 | 5.3 (1.7) | 94.8 (13.2) | World Health Organization criteria | 11 | 21 | 0.5 |

vs., versus; NR, Not Reported, Mos, Months, Mod, Moderate; Sev, Severe; ORT, Oral Rehydration Therapy.

1This study included 5 treatment arms: 1) *L. casei*; 2) *S. boulardii*; 3) *B. clausii*; 4) *L. delbrueckii var bulgaricus*, *L. acidophilus, S. thermophilus, B. bifidum*; 5) *E. faecium.*

†Dehydration Severity represents the severity assessment classification assigned by the study authors.

* Percentage represents the percentage of patients who had vomited prior to enrolment.

- data not reported

1. Canani RB, Cirillo P, Terrin G, et al. Probiotics for treatment of acute diarrhoea in children: randomised clinical trial of five different preparations. BMJ 2007;335:340.

2. Henker J, Laass M, Blokhin BM, et al. The probiotic Escherichia coli strain Nissle 1917 (EcN) stops acute diarrhoea in infants and toddlers. Eur J Pediatr 2007;166:311-8.

3. Henker J, Laass MW, Blokhin BM, et al. Probiotic Escherichia coli Nissle 1917 versus placebo for treating diarrhea of greater than 4 days duration in infants and toddlers. Pediatr Infect Dis J 2008;27:494-9.

4. Nixon AF, Cunningham SJ, Cohen HW, Crain EF. The effect of Lactobacillus GG on acute diarrheal illness in the pediatric emergency department. Pediatr Emerg Care 2012;28:1048-51.

5. Passariello A, Terrin G, Cecere G, et al. Randomised clinical trial: efficacy of a new synbiotic formulation containing Lactobacillus paracasei B21060 plus arabinogalactan and xilooligosaccharides in children with acute diarrhoea. Aliment Pharmacol Ther 2012;35:782-8.

6. Shavit I, Brant R, Nijssen-Jordan C, Galbraith R, Johnson DW. A novel imaging technique to measure capillary-refill time: improving diagnostic accuracy for dehydration in young children with gastroenteritis. Pediatrics 2006;118:2402-8.

7. Friedman JN, Goldman RD, Srivastava R, Parkin PC. Development of a clinical dehydration scale for use in children between 1 and 36 months of age. J Pediatr 2004;145:201-7.

8. Ritchie BK, Brewster DR, Tran CD, Davidson GP, McNeil Y, Butler RN. Efficacy of Lactobacillus GG in aboriginal children with acute diarrhoeal disease: a randomised clinical trial. J Pediatr Gastroenterol Nutr 2010;50:619-24.
