# Supplementary material for: Gastroenteritis Therapies in Developed Countries: Systematic Review and Meta-Analysis
Source: PLoS One. 2015 Jun 15;10(6):e0128754. doi: 10.1371/journal.pone.0128754 (PMC4468143; doi:10.1371/journal.pone.0128754)
Supplement: S5 Table — (DOC) [file pone.0128754.s007.doc]

**S5 Table. Baseline Characteristics - Intravenous Fluids**

| **Study** | **Comparison** | **Country of Study;**  **Years** | **Number of patients** | **Age, Mos: Mean (SD) or Median [IQR]** | **Duration of Symptoms, Hours: Mean (SD)** | **Method of Dehydration Assessment** | **Dehydration Severity†**  **Mean (SD) or Median [IQR]** | **Vomiting %** | **Diarrhea %** |
| --- | --- | --- | --- | --- | --- | --- | --- | --- | --- |
| Allen[^1^](#_ENREF_1) | Plasma-Lyte A vs 0.9% sodium chloride | Canada, US; 2011-13 | 100 | 40.1 | - | Clinical dehydration scale | Dehydration score (Gorelick)[^2^](#_ENREF_2): 5, [5, 6] | - | - |
| Freedman[^3^](#_ENREF_3) | Rapid IV vs Standard IV | Canada;  2006-10 | 226 | 27.6 [15.6-50.4] | - | Clinical dehydration scale[^4^](#_ENREF_4) | Clinical dehydration score (Friedman)[^5^](#_ENREF_5): 4.5 (1.2) | - | - |
| Levy[^6^](#_ENREF_6) | 5% dextrose in normal saline vs. normal saline | US; 2007-10 | 188 | 26.4 [15.0, 42.6] | - | Clinical dehydraiton scale | Dehydration score (Gorelick): 4, [3, 5 | - | - |
| Nager[^7^](#_ENREF_7) | Rapid IV vs Standard IV | USA;  2003-07 | 92 | 13.7 (8.6) | - | Determined using clinical parameters[^8^](#_ENREF_8) | All patients had moderate (6-9%) dehydration (AAP guideline) | 98 | 84 |
| Neville[^9^](#_ENREF_9) | Isotonic IV vs Hypotonic IV | Australia;  2002 | 124 | 29.4 (21) | 36 (NR) | Estimated employing standard clinical measures[^10^](#_ENREF_10) | Percent dehydration: 5% [3% -7%] | 81 | 59 |
| Powell[^11^](#_ENREF_11) | Rapid NG vs Standard NG | Australia;  NR | 254 | 13.2 (1.4) | - | Clinical dehydration scale[^2^](#_ENREF_2) | Dehydration score (Gorelick): 4.8 (0.13) | - | - |

IV, Intravenenous; Mos, Months; NR, Not Reported; American Academy of Pediatrics.

†Dehydration Severity represents the severity assessment classification assigned by the study authors.

- data not reported

1. Allen CH, Goldman RD, Simon HK, et al. Balanced Crystalloid or Saline in Pediatric Gastroenteritis: A Randomized Controlled Trial. SAEM Annual Meeting Abstracts Academic Emergency Medicine, 21: S5–S327 doi: 101111/acem123652014.

2. Gorelick MH, Shaw KN, Murphy KO. Validity and reliability of clinical signs in the diagnosis of dehydration in children. Pediatrics 1997;99:E6.

3. Freedman SB, Parkin PC, Willan AR, Schuh S. Rapid versus standard intravenous rehydration in paediatric gastroenteritis: pragmatic blinded randomised clinical trial. BMJ 2011;343:d6976.

4. Parkin PC, Macarthur C, Khambalia A, Goldman RD, Friedman JN. Clinical and laboratory assessment of dehydration severity in children with acute gastroenteritis. Clin Pediatr (Phila) 2010;49:235-9.

5. Friedman JN, Goldman RD, Srivastava R, Parkin PC. Development of a clinical dehydration scale for use in children between 1 and 36 months of age. J Pediatr 2004;145:201-7.

6. Levy JA, Bachur RG, Monuteaux MC, Waltzman M. Intravenous dextrose for children with gastroenteritis and dehydration: a double-blind randomized controlled trial. Ann Emerg Med 2013;61:281-8.

7. Nager AL, Wang VJ. Comparison of ultrarapid and rapid intravenous hydration in pediatric patients with dehydration. Am J Emerg Med 2010;28:123-9.

8. Practice parameter: the management of acute gastroenteritis in young children. American Academy of Pediatrics, Provisional Committee on Quality Improvement, Subcommittee on Acute Gastroenteritis. Pediatrics 1996;97:424-35.

9. Neville KA, Verge CF, Rosenberg AR, O'Meara MW, Walker JL. Isotonic is better than hypotonic saline for intravenous rehydration of children with gastroenteritis: a prospective randomised study. Arch Dis Child 2006;91:226-32.

10. Adelman RD, Solhaug MJ. Pathophysiology of body fluids and fluid therapy. In: Behrman RE, Kliegman RM, Jenson HB, eds. Nelson textbook of pediatrics. Philadelphia, PA: WB Saunders; 2000:211-15.

11. Powell CV, Priestley SJ, Young S, Heine RG. Randomized clinical trial of rapid versus 24-hour rehydration for children with acute gastroenteritis. Pediatrics 2011;128:e771-8.
